# Supplementary material for: Long Noncoding RNA lncMUMA Reverses Established Skeletal Muscle Atrophy following Mechanical Unloading
Source: Mol Ther. 2018 Oct 25;26(11):2669–80. doi: 10.1016/j.ymthe.2018.09.014 (PMC6225098; doi:10.1016/j.ymthe.2018.09.014)
Supplement: Document S1. Figures S1–S8 and Tables S1 and S2 [file mmc1.pdf]

YMTHE, Volume *26*

Supplemental Information

Long Noncoding RNA lncMUMA Reverses Established Skeletal Muscle Atrophy following Mechanical Unloading

Zong-Kang Zhang, Jie Li, Daogang Guan, Chao Liang, Zhenjian Zhuo, Jin Liu, Aiping Lu, Ge Zhang, and Bao-Ting Zhang

>AK014246
TTTGTACATGACAGTTGTATACCTTTCTCTTGTATCTGACACAAAACCTATGATAAAT
ACTCAAACAATCCCGACTGGATCGAGGAACCATGTATGGGGTAGACTTTTGTATTAA
GAGCCAAGAAAGGGAGCTTACGAATCATGTTTCCTACGTTTTTATACTGTTTGTTTTA
AACGGCAGCCCTGCCCAGTGGGTTCAGCTTTTCTAGGAAGTGAATGTCAGTACTGG
TGTTTTCTATAGGAATGGAGAAGCCATGTATACACTGTGTAAATGCTCATGTGAGAA
TGACCTAGCGGCACAATCTGACTTGCCTTGGCCTCTGGCCTTCCGGTTACTGTTTTT
GGCAGCTCTCTCTACCTTCCTCTATCCTCAAACCTTGTGCCTGTAGCTTTGTACTTC
AGCTCCCAGGGATAGGAACAGACCTAGTGAACATTCCACGGTGCCTGATCTCGCTG
GCAACTGAGTCCAGCTAGGGCCTGACCCAGCGTCAGTCTCCAAAGCTCTGCTTCC
GGATTCCAAACACTGGCGTGAGGGGCAGTAGTCAGCACTTCTAGATCACCATCTAG
TGAGTCGCTGGTGTAGAGTGAACTTTTACTGCACACTAAGGGCTCACAATTAAAATA
AACCAGAATAGCTTTTTGCTCATGGTAACCAAGTTCAGTGTCTGTGGGGCCCACAG
CCTGGCAGGTCTGGCCCAGCTCTCTGTCACCTGCTGTGGGAGATGGACCGTTTGA
CCTCTCTGGAGGCTGAGACCCCATACCTGCTGCTCAGTGCTGGTAATCAGCCCTCC
CCAGAGTGCGTGCCGGCCAGAGGGCTCCACCCACAGTCCCTGGTCATGTACGCAC
ATCACACTCTTCCTGCCTCTGCACTATGAAAACCATACTGGGAGTTTTAGAAGTGCT
CACTCTTGTACGAGTGTCTGCGGACATGTGTAAAATAAACGTTAAACTCTGCTTCG

Figure S1 Sequence of lncMUMA

Table S1 Top 50 down-regulated and top 50 up-regulated lncRNAs in HLS muscle tissues

No.	Gene Name	Fold Change	No.	Gene Name	Fold Change	No.	Gene Name	Fold Change	No.	Gene Name	Fold Change
Top 50 Down-regulated lncRNAs						Top 50 Up-regulated lncRNAs					
1	AK014246 (lncMUMA)	21.7411	26	AK019096	3.950669	1	AK133680	18.32983	26	AK142736	3.894358
2	LOC100046151	12.95444	27	Gm2710	3.931119	2	AK006720	16.05375	27	AK050117	3.80183
3	1600014K23Rik	10.90429	28	Gm8709	3.917914	3	AK010427	14.75385	28	AK080530	3.772047
4	LOC101056557	9.30606	29	AK164498	3.917582	4	AK008861	10.43807	29	AK048321	3.739837
5	AK019626	8.573749	30	AK004808	3.782735	5	AK037838	8.354839	30	AK018045	3.690979
6	AK040479	8.236479	31	AK090094	3.758475	6	AK050666	8.166809	31	AK005231	3.687683
7	AK088055	7.813218	32	AK017133	3.742424	7	AK158068	6.773596	32	AK041461	3.684211
8	AK140828	6.713292	33	AK137267	3.695415	8	AK172445	6.474126	33	AK002372	3.6197
9	AK089627	5.625397	34	AK008597	3.643666	9	AK003280	5.942007	34	AK011222	3.550058
10	LOC636901	5.614903	35	AK038180	3.630421	10	AK172311	5.595007	35	AK012252	3.533306
11	Gm15453	5.529144	36	Gm5396	3.613182	11	AK165502	5.37358	36	AK003389	3.495472
12	Gm10052	5.313084	37	AK141617	3.575356	12	AK003454	5.304729	37	AK003573	3.388748
13	AK137307	5.281147	38	AK156638	3.568095	13	AK013490	5.049814	38	AK172322	3.379735
14	0610009O20Rik	5.116497	39	AK132982	3.560905	14	AK142700	4.890915	39	AK135737	3.37448
15	LOC100045968	4.969623	40	Gm6498	3.549732	15	AK172626	4.823841	40	AK162281	3.347527
16	AK032367	4.934769	41	AK043296	3.522517	16	AK004150	4.691858	41	AK013679	3.311203
17	AK138117	4.755483	42	Gm5506	3.476566	17	AK002796	4.390714	42	AK087626	3.261159
18	AK075592	4.485645	43	Gm13277	3.436207	18	AK038876	4.381345	43	AK136079	3.226031
19	AK033909	4.429835	44	AK039307	3.397279	19	AK002224	4.353103	44	AK136295	3.191652
20	AK087321	4.341787	45	AK036353	3.363755	20	AK011347	4.26089	45	AK021173	3.167185
21	AK146676	4.259343	46	AK030937	3.34493	21	AK014372	4.121564	46	AK046094	3.165406
22	AK028893	4.205192	47	AK050744	3.313224	22	AK003535	4.115442	47	AK131727	3.142544
23	AK088710	4.180129	48	AK156875	3.288136	23	AK014142	4.054426	48	AK144859	3.138059
24	AK157223	4.115882	49	Gm5331	3.262516	24	AK005641	4.001631	49	AK013681	3.101982
25	AK034076	4.070582	50	AK081791	3.238565	25	AK084371	3.95384	50	AK016405	3.094581

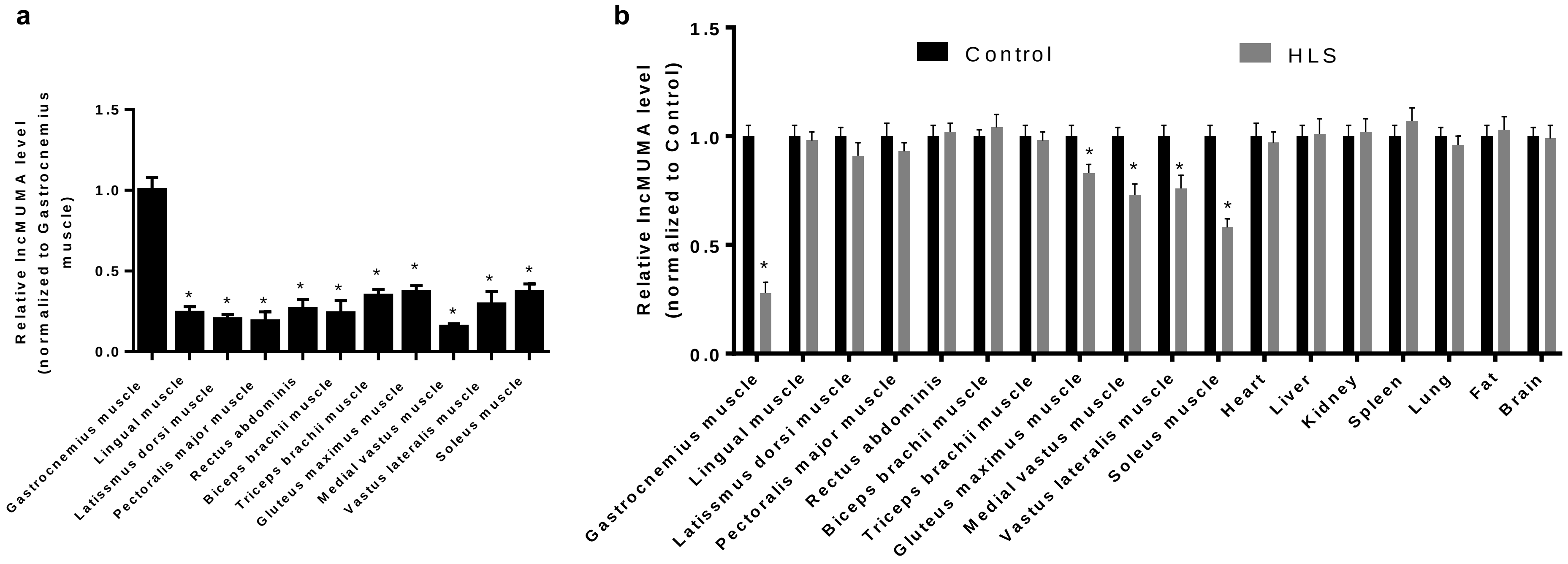

Figure S2 LncMUMA was highly expressed in gastrocnemius muscle and didn't significantly alter in forelimb muscles and other tissue/organs after HLS. (**a**) Real-time PCR analysis of lncMUMA levels in different skeletal muscles of mice. * *P*<0.05 vs. Gastrocnemius muscle. (**b**) Real-time PCR analysis of lncMUMA levels in different skeletal muscles and other tissue/organs of mice with or without HLS treatment. n=10. Data are presented as mean ± SEM. * *P*<0.05 vs. Control.

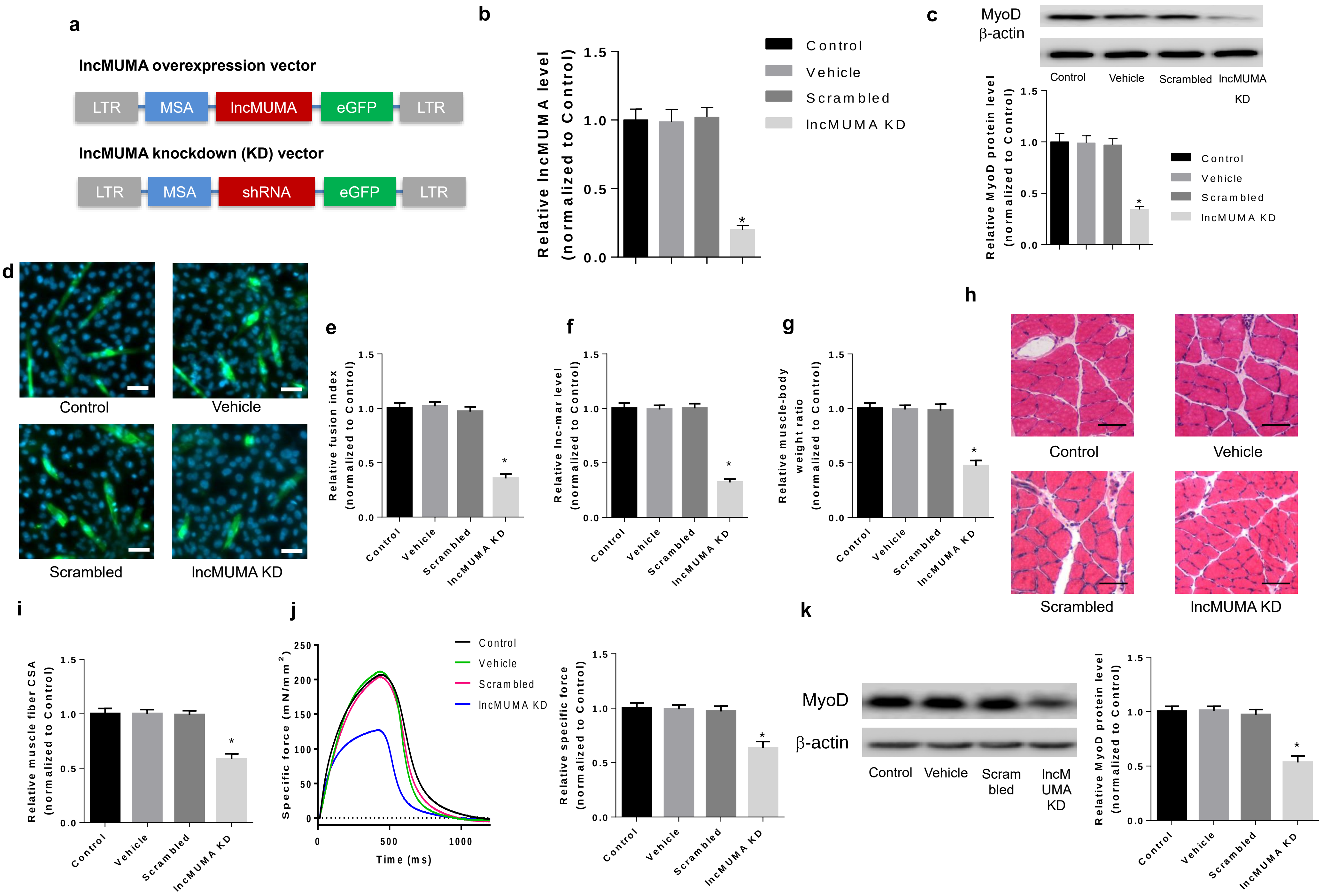
a
lncMUMA overexpression vector
LTR
MSA
lncMUMA
eGFP
LTR
lncMUMA knockdown (KD) vector
LTR
MSA
shRNA
eGFP
LTR
b
Relative lncMUMA level (normalized to Control)
Control
Vehicle
Scrambled
lncMUMA KD
c
MyoD
β-actin
Control
Vehicle
Scrambled
lncMUMA KD
Relative MyoD protein level (normalized to Control)
d
Control
Vehicle
Scrambled
lncMUMA KD
e
Relative fusion index (normalized to Control)
f
Relative lnc-mar level (normalized to Control)
g
Relative muscle-body weight ratio (normalized to Control)
h
Control
Vehicle
Scrambled
lncMUMA KD
i
Relative muscle fiber CSA (normalized to Control)
j
Specific force (mN/mm²)
Time (ms)
Relative specific force (normalized to Control)
k
MyoD
β-actin
Control
Vehicle
Scram bled
lncM UMA KD
Relative MyoD protein level (normalized to Control)

Figure S3 LncMUMA silencing decreased the myogenesis in C2C12 myoblasts *in vitro* and decreased muscle mass/structure/strength in adult mice. (a) Schematic of lncMUMA overexpression and knockdown lentiviral vector for viral preparation, respectively. (**b**) Real-time PCR analysis of lncMUMA levels in C2C12 cells transduced with lentivirus constructed with either scrambled shRNA or lncMUMA knockdown (KD) vector on Day 7 of differentiation under normal gravity culture environment. (**c**) Western blot analysis of MyoD protein level in lentivirus transduced C2C12 cells on Day 7 of differentiation under normal gravity culture environment. (**d**) Representative images of C2C12 cells transduced with lentivirus on Day 7 of differentiation under normal gravity culture environment. Myosin was labeled with green fluorescence, and the nuclei were labeled with DAPI. Scale bar=50 μm. (**e**) The fusion index in lentivirus transduced C2C12 cells on Day 7 of differentiation under normal gravity culture environment. (**f**) Real-time PCR analysis of lncMUMA level in soleus muscle of mice treated with lentivirus with EV and lncMUMA KD vector, respectively. (**g-i**) Gastrocnemius muscle-to-body weight ratio (**g**), cross-sections from mid-belly gastrocnemius muscle (**h**) and muscle fiber CSA (**i**) in mice treated with lentivirus with scrambled shRNA and lncMUMA KD vector, respectively. Scale bar= 50 μm. (**l**) *In vitro* muscle function testing of specific force in gastrocnemius muscle of mice treated with lentivirus with scrambled shRNA and lncMUMA KD vector, respectively. (**J**) Expression of MyoD protein in gastrocnemius muscle of mice treated with lentivirus with scrambled shRNA and lncMUMA KD vector, respectively. n=5 for *in vitro* and n=10 for *in vivo*. U6 small nuclear RNA is used as the endogenous control of lncRNA. β-actin is used as the endogenous control for protein. Data are presented as mean ± SEM. * $P<0.05$ vs. Control.

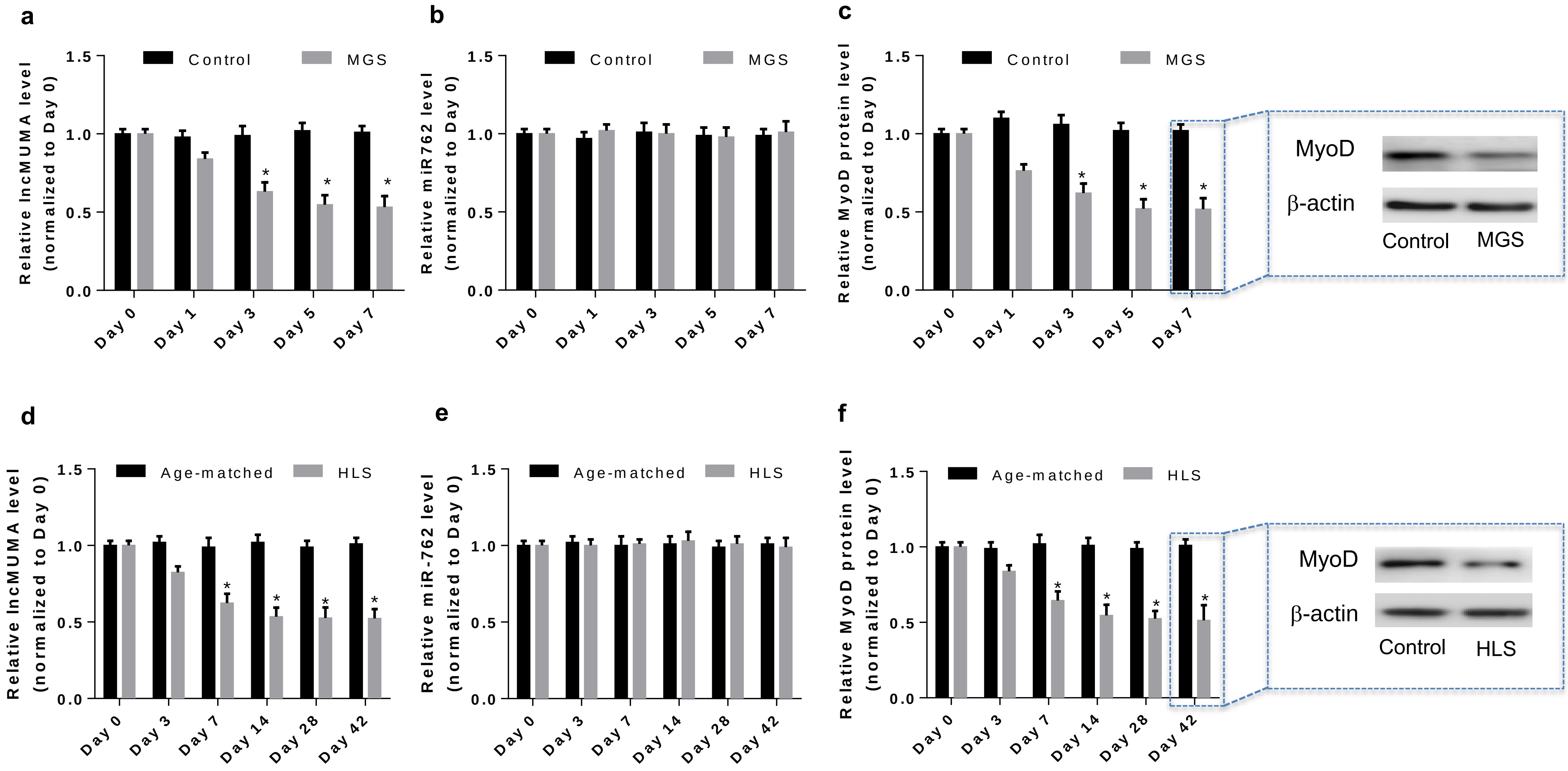

Figure S4 Reduced MyoD expression associated with decreased lncMUMA expression, without changes of miR-762, in microgravity-simulated (MGS) C2C12 myoblasts *in vitro* and in muscle tissues of hindlimb suspension (HLS) mice. (**a**) Real-time PCR analysis of lncMUMA levels in C2C12 cells with either normal gravity (Control) or MGS culture environment on Day 0, 1, 3, 5 and 7 of differentiation. (**b**) Expression level of miR-762 in C2C12 cells of each group at each time point. (**c**) (**left**) Expression level of MyoD protein in C2C12 cells of each group at each time point. (**right**) Representative western blot images of MyoD protein in C2C12 cells on Day 7 of differentiation. (**d**) Expression level of lncMUMA in gastrocnemius muscle of either healthy adult (Control) or HLS mice on Day 0, 3, 7, 14, 28 and 42 of HLS. (**e**) Expression level of miR-762 in gastrocnemius muscle of each group at each time point. (**f**)(**left**) Expression level of MyoD protein in gastrocnemius muscle of each group at each time point. (**right**) Representative western blot images of MyoD protein in gastrocnemius muscle of either Control or HLS mice on Day 42 of HLS. n=5 (*in vitro*) and 10 (*in vivo*) at each time point for each group. U6 small nuclear RNA is used as the internal control of lncRNA and miRNA. β-actin is used as the control for protein. Data are presented as mean ± SEM. *P<0.05 vs. Corresponding Day 0.

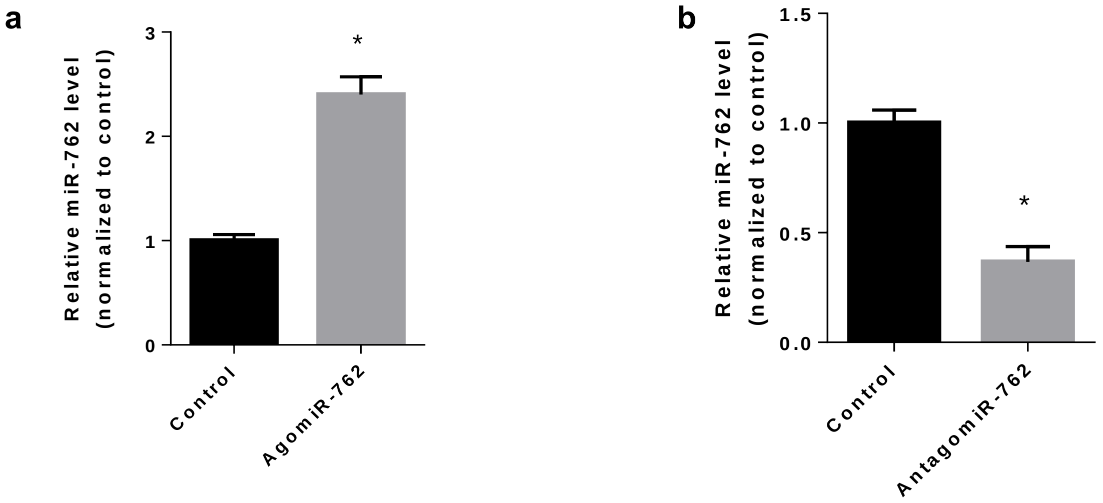

Figure S5 AgomiR-762 and AntagomiR-762 regulated the expression level of miR-762 in C2C12 myoblasts *in vitro*. Real-time PCR analysis of miR-762 level in C2C12 cells treated with AgomiR-762 (**a**) and AntagomiR-762 (**b**). U6 small nuclear RNA is used as the endogenous control. Data are presented as mean ± SEM. * *P*<0.05 vs. Control.

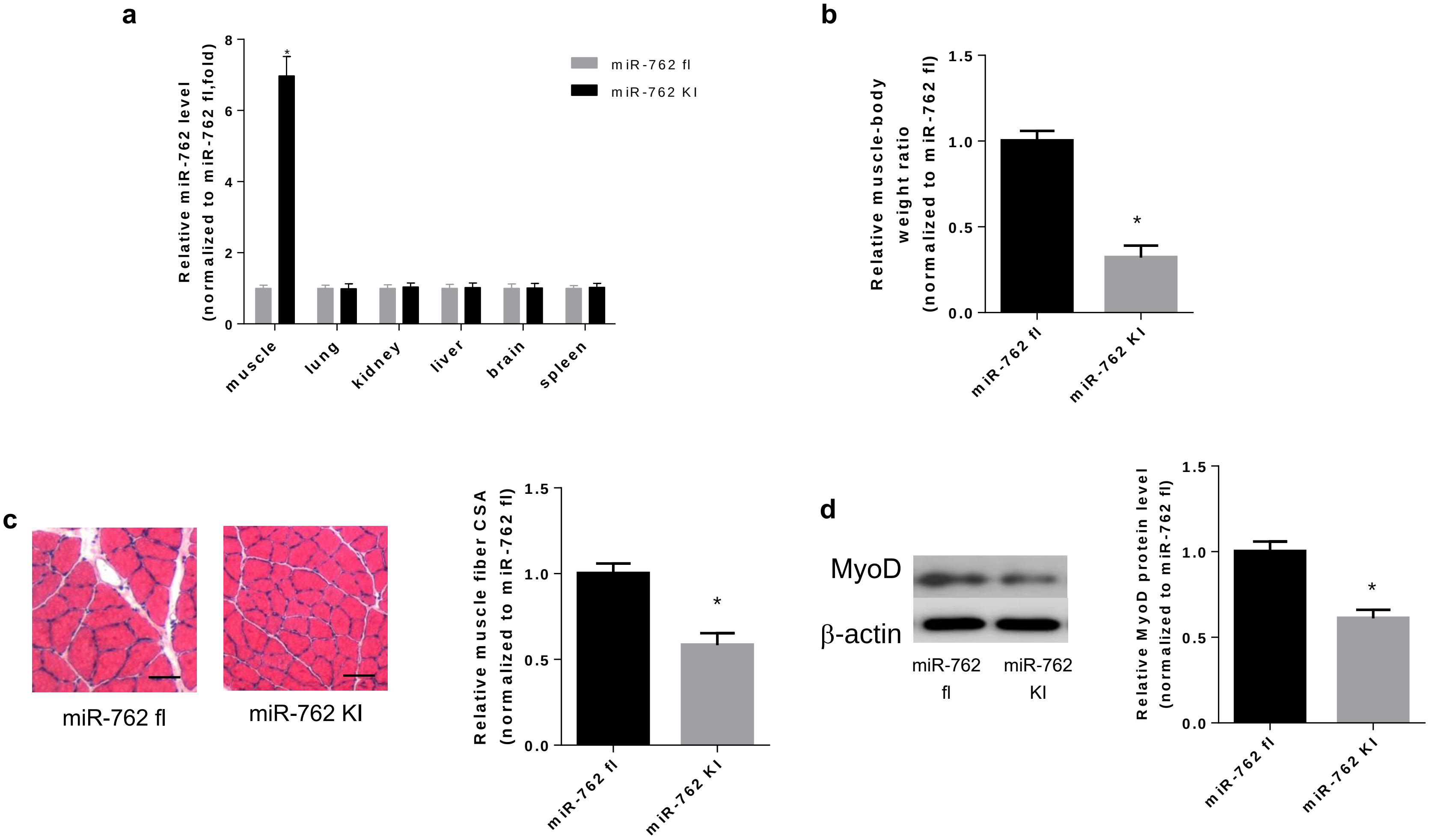

Figure S6 Phenotype analysis of MyoD expression, muscle mass/structure/function in muscle-specific miR-762 knockin mice. (a) Real-time PCR analysis showing the miR-762 expression in muscle, lung, kidney, liver, brain and spleen tissues isolated from ROSA26-PCAG-STOP^{flox}-*miR-762*-eGFP (miR-762 fl) and miR-762 knockin (miR-762 KI) mice, respectively. **(b)** Gastrocnemius muscle-to-body weight ratio in miR-762 fl and miR-762 KI mice, respectively. (**c**) Cross-sections from mid-belly gastrocnemius muscle and muscle fiber CSA in miR-762 fl and miR-762 KI mice, respectively. Scale bar= 50μm. (**d**) Expression level of MyoD protein in gastrocnemius muscle of miR-762 fl and miR-762 KI mice, respectively. n=10 for each group. U6 small nuclear RNA is used as the endogenous control of miRNA. β-actin is used as the endogenous control for protein. Data are presented as mean ± SEM. **P*<0.05 vs. miR-762 fl group.

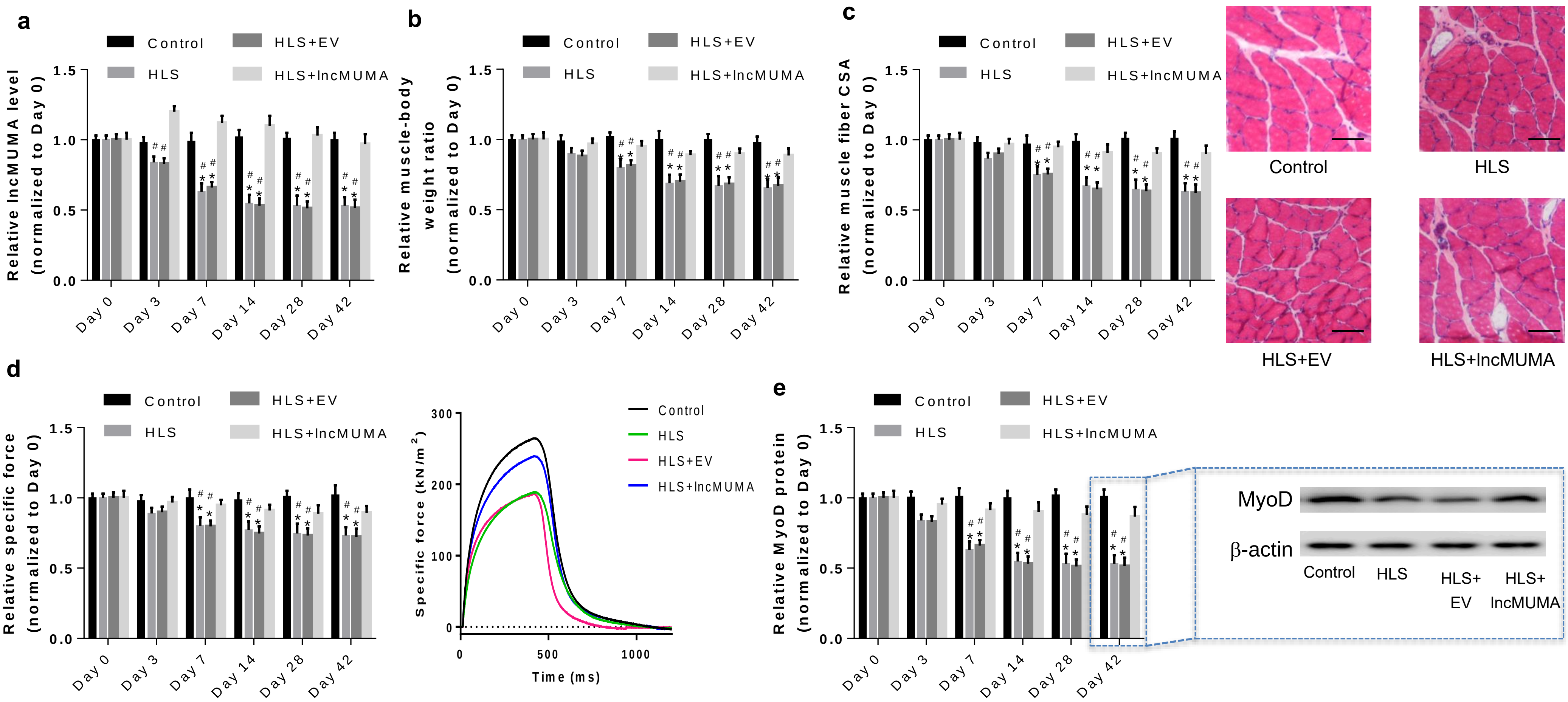

Figure S7 Skeletal muscle-specific overexpression of lncMUMA before unloading attenuated the decreases of MyoD, muscle mass/structure/function from baseline following mechanical unloading. (**a**) Real-time PCR analysis of lncMUMA levels in gastrocnemius muscle of normal control, hindlimb suspension (HLS), HLS+empty vector (EV) and HLS+lncMUMA vector (lncMUMA) mice, respectively, on Day 0, 3, 7, 14, 28 and 42 of HLS. (**b**) Gastrocnemius muscle-to-body weight ratio in each group at each time point. (**c**) (**left**) Gastrocnemius muscle fiber CSA in each group at each time point. (**right**) Cross-sections from mid-belly gastrocnemius muscle in each group at Day 42 of HLS. Scale bar= 50μm. (**d**) (**left**) *In situ* muscle function testing of specific force in gastrocnemius muscle of each group at each time point. (**right**) Specific force in gastrocnemius muscle of each group at Day 42 of HLS. (**e**) (**left**) Expression level of MyoD protein in each group at each time point. (**right**) Expression level of MyoD protein in gastrocnemius muscle of each group at Day 42 of HLS. n=10 at each time point for each group. U6 small nuclear RNA is used as the internal control of lncRNA. β-actin is used as the control for protein. Data are presented as mean ± SEM. * *P*<0.05 vs. Control, # *P*<0.05 vs. HLS+lncMUMA.

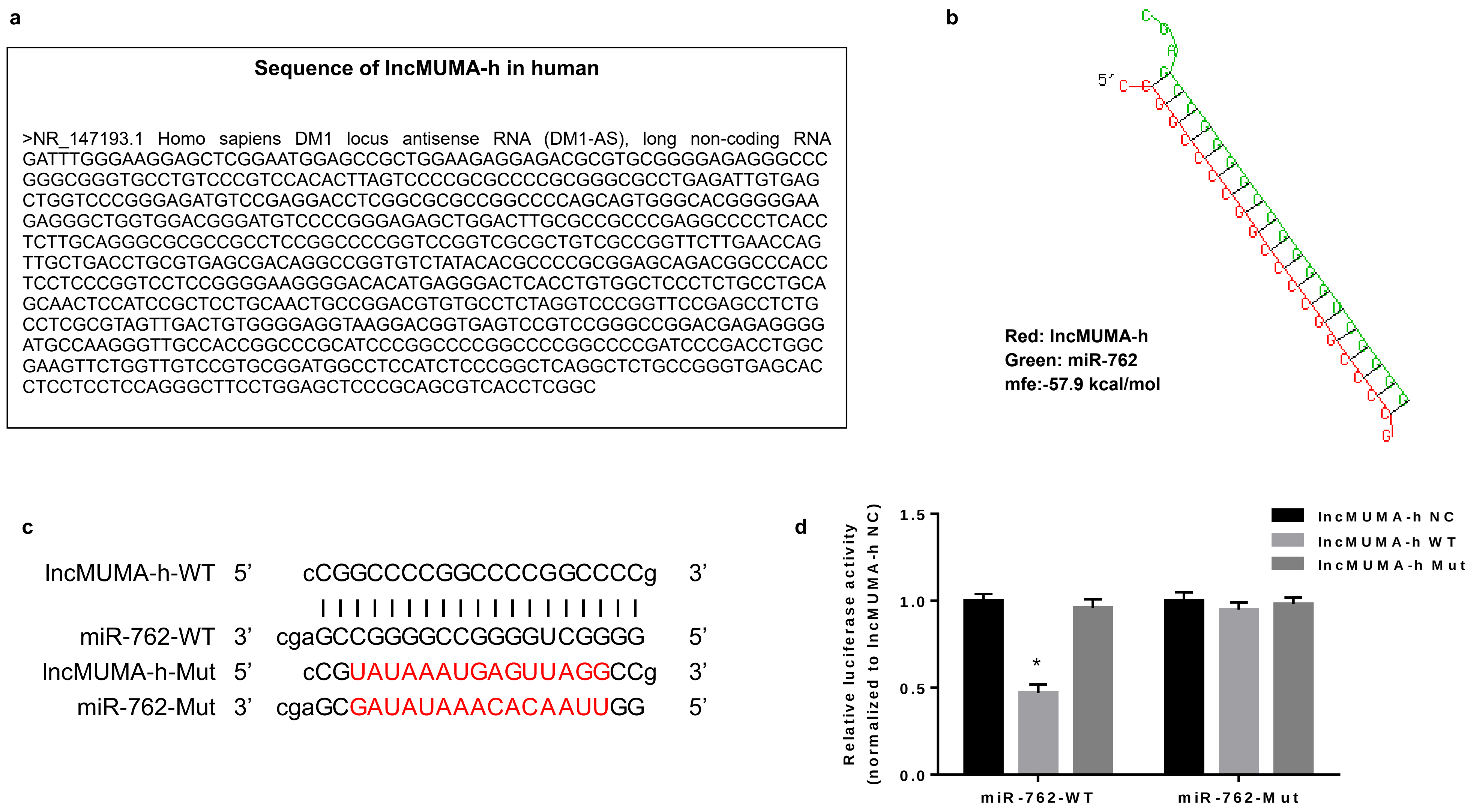

Figure S8 lncMUMA was functionally conserved in primary human skeletal muscle cells (pHSkMC). (**a**) Sequence of lncMUMA in human (lncMUMA-h). (**b**) Bioinformatic prediction of miR-762 as a target miRNA of lncMUMA-h by RNAhybrid 2.12. mfe: minimum free energy. (**c**) Sequence of wild type and mutated binding site between miR-762 and lncMUMA-h. (**d**) Luciferase reporter assay of either wild type miR-762-transfected (miR-762-WT) or mutated miR-762-transfected (miR-762-Mut) pHSkMCs from healthy adults treated with negative control (lncMUMA-h NC), wild type binding site of lncMUMA (lncMUMA-h WT) and mutated binding site of lncMUMA (lncMUMA-h Mut), respectively. n=3. * $P<0.05$ vs. lncMUMA-h NC. Data are presented as mean ± SEM.

Table S2 Primer sequences of lncRNA, miRNA and mRNA

Gene	Primer sequences
lncMUMA	forward: TTGTACTTCAGCTCCCAGGG<br>reverse: CCAGCGACTCACTAGATGGT
miR-762	forward: ACACGGGGCTGGGGCCGGGGCCGAGCGCCTC<br>reverse: CTCAGGGGCTGGGGCCGGGGCCGAGCCAGA
Myod1	forward: AGTGAATGAGGCCTTCGAGA<br>reverse: GCATCTGAGTCGCCACTGTA
