# Supplementary material for: Long Noncoding RNA lncMUMA Reverses Established Skeletal Muscle Atrophy following Mechanical Unloading
Source: Mol Ther. 2018 Oct 25;26(11):2669–80. doi: 10.1016/j.ymthe.2018.09.014 (PMC6225098; doi:10.1016/j.ymthe.2018.09.014)
Supplement: Document S2. Article plus Supplemental Information [file mmc2.pdf]

Molecular Therapy
Original Article

Long Noncoding RNA lncMUMA Reverses Established Skeletal Muscle Atrophy following Mechanical Unloading

Zong-Kang Zhang,[1,4] Jie Li,[1,4] Daogang Guan,[2,4] Chao Liang,[3,4] Zhenjian Zhuo,[1] Jin Liu,[3] Aiping Lu,[2] Ge Zhang,[3] and Bao-Ting Zhang[1]

[1]School of Chinese Medicine, Faculty of Medicine, The Chinese University of Hong Kong, Shatin, New Territories, Hong Kong SAR, China; [2]Institute of Integrated Bioinformedicine and Translational Science, School of Chinese Medicine, Hong Kong Baptist University, Kowloon Tong, Kowloon, Hong Kong SAR, China; [3]Institute for Advancing Translational Medicine in Bone & Joint Diseases, School of Chinese Medicine, Hong Kong Baptist University, Kowloon Tong, Kowloon, Hong Kong SAR, China

Reversing established muscle atrophy following mechanical unloading is of great clinical challenge. Long noncoding RNAs (lncRNAs) have been demonstrated to play important roles in myogenesis. Here we identified a lncRNA (mechanical unloading-induced muscle atrophy-related lncRNA [lncMUMA]) enriched in muscle, which was the most downregulated lncRNA during muscle atrophy development in hindlimb suspension (HLS) mice. The *in vitro* and *in vivo* data demonstrated that the decreased expression levels of lncMUMA closely associated with a reduction of myogenesis during mechanical unloading. Mechanistically, lncMUMA promoted myogenic differentiation by functioning as a miR-762 sponge to regulate the core myogenic regulator MyoD *in vitro*. The enforced expression of lncMUMA relieved the decreases in MyoD protein and muscle mass in miR-762 knockin mice. Therapeutically, the enforced expression of lncMUMA improved the *in vitro* myogenic differentiation of myoblasts under microgravity simulation, prevented the muscle atrophy development, and reversed the established muscle atrophy in HLS mice. These findings identify lncMUMA as an anabolic regulator to reverse established muscle atrophy following mechanical unloading.

INTRODUCTION

Mechanical unloading induces obvious skeletal muscle atrophy and functional deficit, especially under microgravity environment during spaceflight. Exposure to microgravity for 3–6 months induces skeletal muscle atrophy with more than 40% impairment of functional properties in astronauts during spaceflight.[1,2] Decreased muscle differentiation and protein synthesis are largely responsible for the mechanical unloading-induced skeletal muscle atrophy.[3,4] However, to date there are no effective countermeasures to combat the mechanical unloading-induced skeletal muscle atrophy. Exercises are used to replace mechanical loads during spaceflight. Astronauts on the international space station spend 5 hr/week for aerobic exercise and 3–6 days/week for resistance exercise;[5] the exercises are insufficient to prevent loss of muscle strength and endurance.[2]

For the pharmaceutical approaches, intake of essential amino acids has been proven to increase muscle protein synthesis in astronauts;[6] but, there is concern that increasing amino acid intake during spaceflight may increase bone resorption.[7] Other anabolic agents, such as insulin-like growth factor-I (IGF-I) treatment[8] and anabolic androgenic steroids,[9] have some concerns on increased risks (drug resistance and cardiac events), limiting their therapeutic use for muscle atrophy.[10] Thus, it is desirable to investigate the molecular mechanisms of the compromised muscle anabolism during unloading-induced skeletal muscle atrophy to develop a promising muscle anabolic strategy.

Long noncoding RNAs (lncRNAs) (>200 nt) are implicated as important regulators in numerous physiological and pathological processes.[11] lncRNAs also have been demonstrated to play important roles in myogenesis. The lncRNA H19 can positively regulate the progression of myogenic differentiation by repressing the expression of insulin-like growth factor 2 (IGF2) that negatively regulates the key myogenic regulatory factor MyoD expression.[12] Moreover, H19 also antagonizes microRNA let-7 that impairs insulin signaling and decreases glucose uptake in skeletal muscle, where H19 depletion results in premature muscle differentiation.[13] The competing mode for microRNAs is also found in another lncRNA linc-MD1, which positively correlates with the muscle differentiation.[14] The lncRNA lnc-mg promotes myogenesis by functioning as a competing endogenous RNA (ceRNA) that binds to microRNA-125b to elevate protein

Received 20 March 2018; accepted 14 September 2018;
https://doi.org/10.1016/j.ymthe.2018.09.014.

[4]These authors contributed equally to this work.

Correspondence: Bao-Ting Zhang, School of Chinese Medicine, Faculty of Medicine, The Chinese University of Hong Kong, Shatin, New Territories, Hong Kong SAR, China.
E-mail: zhangbaoting@cuhk.edu.hk
Correspondence: Ge Zhang, Institute for Advancing Translational Medicine in Bone & Joint Diseases, School of Chinese Medicine, Hong Kong Baptist University, Kowloon Tong, Kowloon, Hong Kong SAR, China.
E-mail: zhangge@hkbu.edu.hk

This is an open access article under the CC BY license (http://creativecommons.org/licenses/by/4.0/).

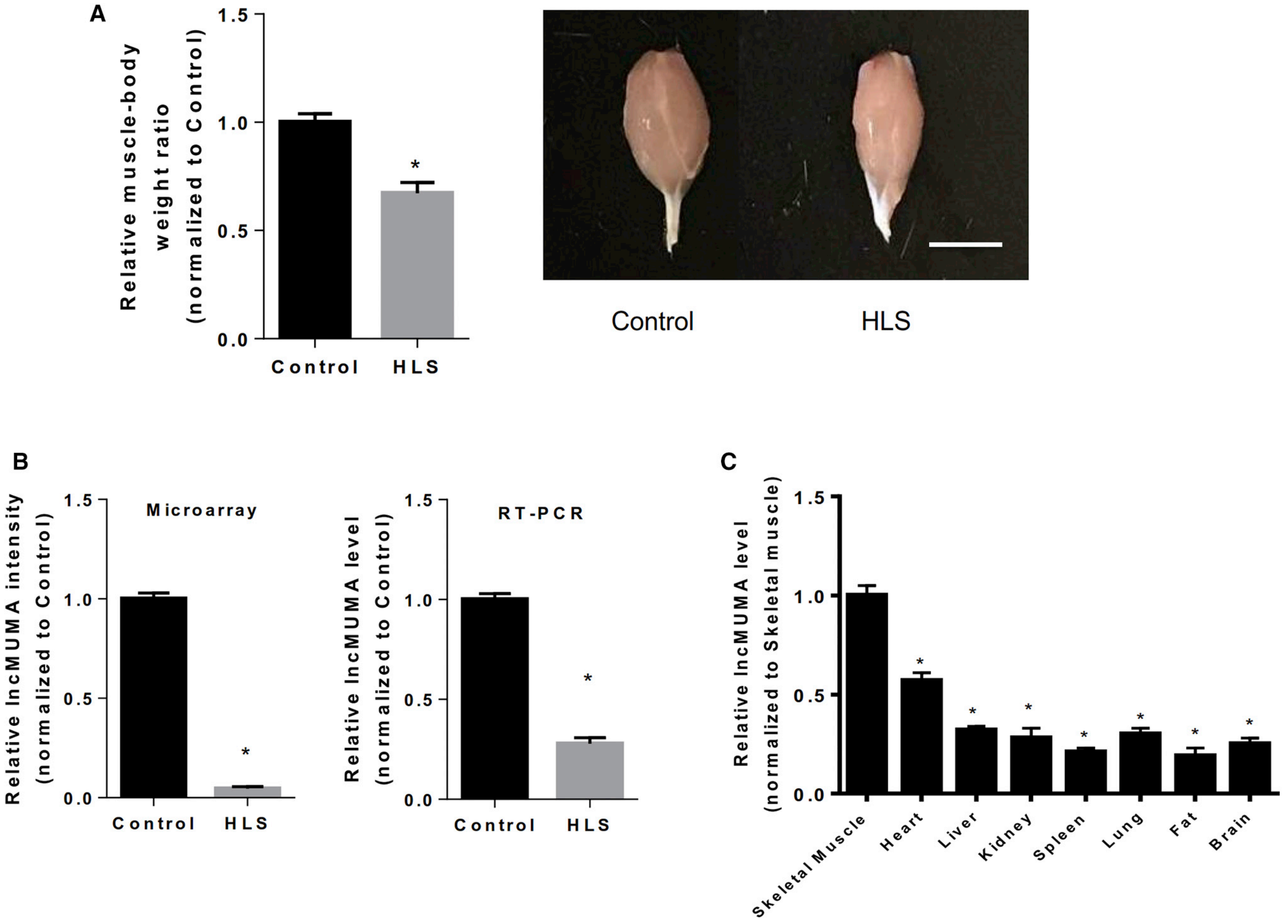

Figure 1. A Newly Identified lncRNA (lncMUMA) Was the Most Downregulated lncRNA in Gastrocnemius Muscle from Hindlimb Suspension Mice

(A) (Left) Gastrocnemius muscle-to-body weight ratio of age-matched control and hindlimb suspension (HLS) mice (n = 3). (Right) Representative images show gastrocnemius muscle from age-matched control and HLS mice. Scale bar, 5 mm. (B) Microarray (left) and RT-PCR analysis (right) of lncMUMA levels in gastrocnemius muscle from normal and HLS mice. (C) Real-time PCR analysis of lncMUMA levels in different tissues and organs of mice. The fat tissue was collected from the abdominal cavity (n = 10). $*p < 0.05$ versus skeletal muscle. U6 is used as the internal control. $*p < 0.05$ versus gastrocnemius muscle. Data are presented as mean ± SEM.

abundance of IGF2.[15] The inhibition of lncRNA Malat1 by myostatin is associated with decreased myogenesis.[16] The lncRNA Yam-1 is downregulated upon differentiation and acts as an inhibitor of myogenesis through in *cis* regulation of miR-715, which in turn targets Wnt7b to repress myogenesis.[17] However, the specific roles of lncRNAs in muscle atrophy following mechanical unloading are still largely unknown. Thus, the aim of our study was to explore the role of lncRNAs in regulating muscle differentiation during the skeletal muscle atrophy following mechanical unloading.

In this study, we identified a lncRNA (mechanical unloading-induced muscle atrophy-related lncRNA [lncMUMA]) in mice skeletal muscle. The decreased expression level of lncMUMA was closely associated with a reduction of myogenesis during mechanical unloading *in vitro* and *in vivo*. Mechanistically, we demonstrate that lncMUMA functioned as a miR-762 sponge to regulate the core myogenic regulator MyoD *in vitro* and *in vivo*. Furthermore, we show that the enforced expression of lncMUMA improved the *in vitro* myogenic differentiation of myoblasts under microgravity simulation, prevented the muscle atrophy development, and reversed the established muscle atrophy in hindlimb suspension (HLS) mice. These findings could provide a novel strategy for the treatment of muscle atrophy following mechanical unloading.

RESULTS

lncMUMA Was the Most Downregulated lncRNA in Gastrocnemius Muscle from HLS Mice

We utilized lncRNA microarray to compare the differentially expressed lncRNAs in the gastrocnemius muscle between control and HLS mice (n = 3). The muscle mass was significantly decreased in HLS mice (Figure 1A). A 959-nt-long lncRNA (AK014246) was identified as the most downregulated lncRNA in the gastrocnemius muscle from HLS mice (Figure 1B; Figure S1; Table S1). Further real-time

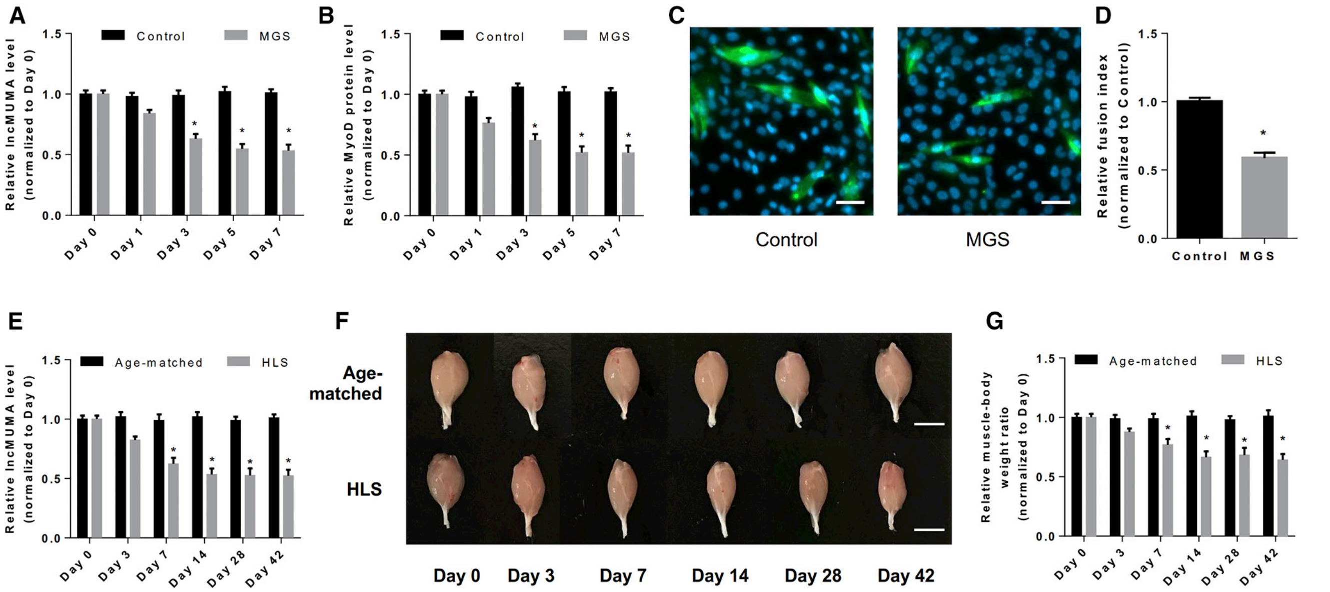

Figure 2. lncMUMA Expression Associated with Muscle Differentiation in Microgravity-Simulated C2C12 Myoblasts *In Vitro* and Muscle Mass in HLS Mice
(A) Real-time PCR analysis of lncMUMA levels in C2C12 cells with either normal gravity (control) or microgravity-simulated (MGS) culture environment on days 0, 1, 3, 5, and 7 of differentiation. (B) Western blot analysis of MyoD protein level in C2C12 cells with either normal gravity (control) or MGS culture environment on days 0, 1, 3, 5, and 7 of differentiation. (C) Representative images of C2C12 cells with either normal gravity (control) or MGS culture environment on day 7 of differentiation. Myosin was labeled with green fluorescence and the nuclei were labeled with DAPI. Scale bars, 50 μm. (D) The fusion index in C2C12 cells with either normal gravity (control) or MGS culture environment on day 7 of differentiation. (E) Real-time PCR analysis of lncMUMA levels in gastrocnemius muscle of either age-matched control or HLS mice. (F and G) Representative images (F) and muscle mass (G) of gastrocnemius muscle from age-matched control and HLS mice during unloading. Scale bar, 5 mm. n = 5 for *in vitro* and n = 10 for *in vivo*. U6 small nuclear RNA is used as the endogenous control of lncRNA. β-actin is used as the endogenous control for protein. Data are presented as mean ± SEM. *p < 0.05 versus the corresponding day 0.

PCR showed that the expression level of the lncRNA was much higher in the skeletal muscle than in other tissues and organs in mice (n = 10) (Figure 1C). Among different skeletal muscles, this lncRNA was the most abundant in gastrocnemius muscle (Figure S2A). Except in the skeletal muscle in hindlimb, the expression levels of the lncRNA in other tissues and organs or skeletal muscles in the forelimb and trunk were not significantly altered during HLS (Figure S2B). The lncRNA was named lncMUMA.

lncMUMA Expression Was Associated with Muscle Differentiation in Microgravity-Simulated C2C12 Myoblasts *In Vitro* and Muscle Mass in HLS Mice

We cultured C2C12 cells in a microgravity-simulated (MGS) environment. Interestingly, the lncMUMA expression was found dramatically decreased and reached a stable low level in the MGS group on day 5 of differentiation. The similar trend was also observed in the expression level of the myogenic marker MyoD protein in the MGS group. Consistently, simulated microgravity also suppressed myotube formation in C2C12 cells, evidenced by a much lower fusion index in the MGS group compared to the control group on day 7 of the differentiation (Figures 2A–2D). Consistent with the *in vitro* findings, we also found that the lncMUMA expression rapidly decreased in the gastrocnemius muscle of mice that underwent HLS, and it reached a stable low level on day 14 of HLS (n = 10) (Figure 2E). A similar trend was also observed in the change of muscle mass in HLS mice (Figures 2F and 2G).

lncMUMA Silencing Suppressed Muscle Differentiation in C2C12 Myoblasts and Decreased Muscle Mass and Strength in Adult Mice

To evaluate the biology function of lncMUMA *in vitro* and *in vivo*, a skeletal muscle-specific lentiviral vector system was constructed with either lncMUMA or lncMUMA short hairpin RNA (shRNA), and the lentivirus for cell and tissue transduction was subsequently prepared (Figure S3A). We transduced C2C12 cells with lncMUMA shRNA lentivirus under normal gravity. The MyoD protein level dramatically decreased in the lncMUMA shRNA group compared to that in the scrambled shRNA group. Consistently, skeletal muscle-specific lncMUMA silencing suppressed myotube formation in C2C12 cells, evidenced by a lower fusion index in the lncMUMA shRNA group compared to the scrambled shRNA group on day 7 of the differentiation (Figures S3B–S3D). The lncMUMA knockdown (KD) lentivirus was injected into the gastrocnemius muscle of adult mice (n = 10). The muscle mass and muscle fiber cross-sectional area (CSA) significantly decreased in the lncMUMA KD group compared to those in the scrambled shRNA group. Consistently, the muscle-specific force and the MyoD protein level were also reduced in the lncMUMA KD group compared to those in the scrambled shRNA group (Figures S3E–S3J).

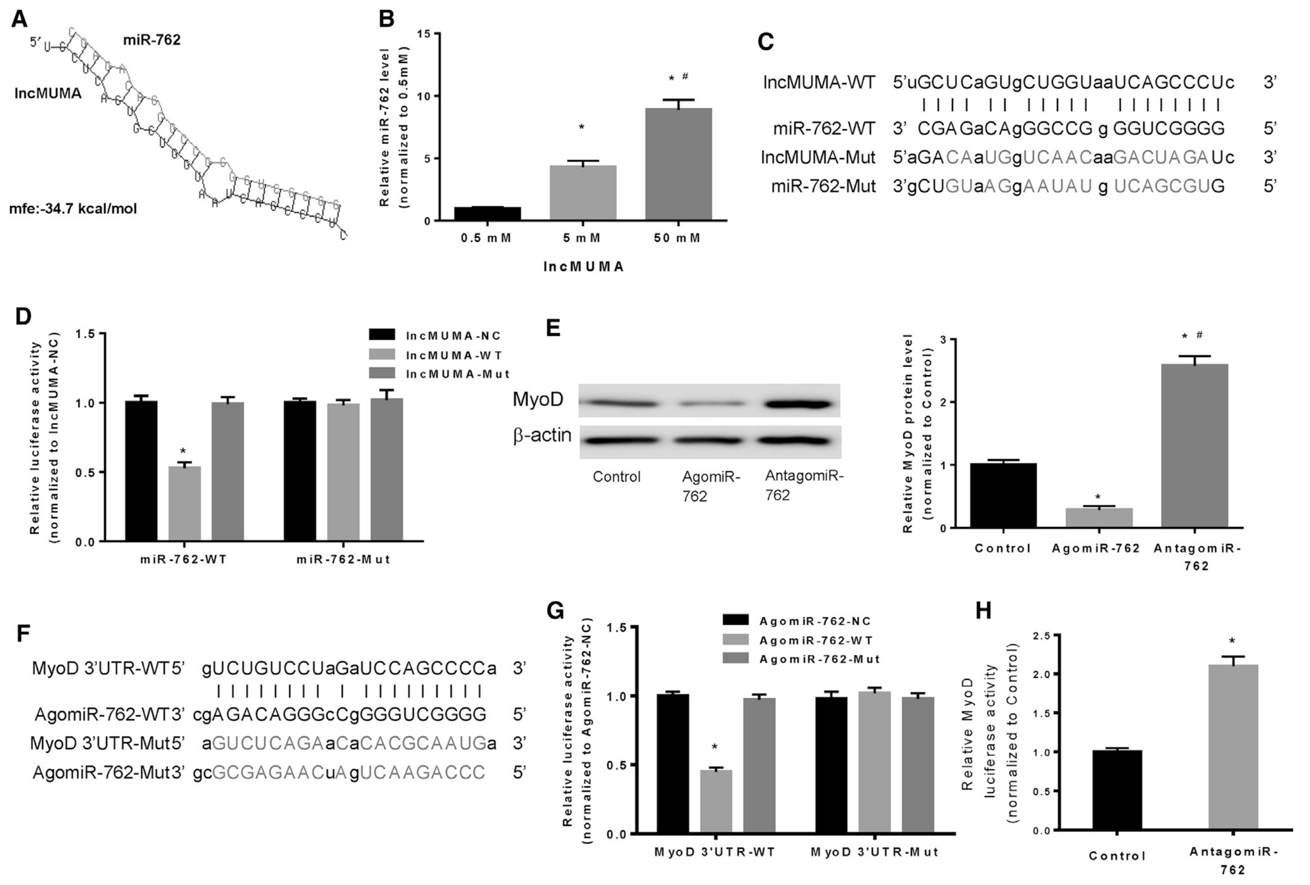

Figure 3. Validation of the Binding of lncMUMA with miR-762 and miR-762 with MyoD in C2C12 Cells *In Vitro*

(A) Bioinformatic prediction of miR-762 as a target miRNA of lncMUMA by RNAhybrid 2.12. mfe, minimum free energy. (B) Pull-down assay combined with real-time PCR analysis of miR-762 level in C2C12 cells transfected with biotin-labeled lncMUMA at different dosages. *p < 0.05 versus 0.5 mM, #p < 0.05 versus 5 mM. (C) Sequence of wild-type and mutated binding site between miR-762 and lncMUMA. (D) Luciferase reporter assay of either wild-type miR-762-transfected (miR-762-WT) or mutated miR-762-transfected (miR-762-Mut) C2C12 cells treated with negative control (lncMUMA-NC), wild-type binding site of lncMUMA (lncMUMA-WT), and mutated binding site of lncMUMA (lncMUMA-Mut), respectively. *p < 0.05 versus lncMUMA-NC. (E) Western blot analysis of expression level of MyoD in C2C12 cells treated with either agomiR-762 or antagomiR-762. *p < 0.05 versus control, #p < 0.05 versus agomiR-762. (F) Sequence of wild-type and mutated binding site between miR-762 and 3′ UTR of MyoD. (G) Luciferase reporter assay of MyoD 3′ UTR and MyoD 3′ UTR-Mut in C2C12 cells transfected with either miR-762-WT or agomiR-762-Mut. *p < 0.05 versus agomiR-762-NC. (H) Luciferase reporter assay of MyoD 3′ UTR in C2C12 cells treated with antagomiR-762. *p < 0.05 versus control. n = 5 for each group. U6 small nuclear RNA is used as the internal control of lncRNA and miRNA. β-actin is used as the internal control for protein. Data are presented as mean ± SEM.

lncMUMA Interacted with miR-762- and miR-762-Targeted MyoD in C2C12 Cells

To gain further insight into the mechanism by which lncMUMA regulates muscle differentiation, we predicted microRNA-762 (miR-762) was one of the target microRNAs (miRNAs) of lncMUMA by using RNAhybrid 2.12 (https://bibiserv.cebitec.uni-bielefeld.de/rnahybrid/).[18] The binding site of lncMUMA to miR-762 is located in 767–789 nt from the 5′ end of lncMUMA (Figure 3A). We found that the expression level of miR-762 was unchanged, while MyoD protein expression significantly decreased in MGS C2C12 cells, which was closely associated with the decreased lncMUMA expression (Figures S4A–S4C). Moreover, consistent data were also found in the gastrocnemius muscle of HLS mice (n = 10) (Figures S4D–S4F).

To further validate the interaction between lncMUMA and miR-762, pull-down assay and luciferase reporter assay were performed. The biotin-labeled lncMUMA specifically pulled down miR-762 in a dose-dependent manner in miR-762-transfected C2C12 cells (Figure 3B). The miR-762 containing either wild-type binding site (miR-762-WT) or mutated binding site (miR-762-Mut) was cloned into the downstream of luciferase reporter gene (Figure 3C). It showed that lncMUMA transduction could reduce the luciferase activity of miR-762-WT, but not affect that of miR-762-Mut. Meanwhile, transduction of lncMUMA containing a mutated binding site could not reduce the luciferase activity of either miR-762-WT or miR-762-Mut (Figure 3D). Moreover, MyoD was downregulated at the protein level by agomiR-762 (a miR-762 agonist) in C2C12 cells, whereas

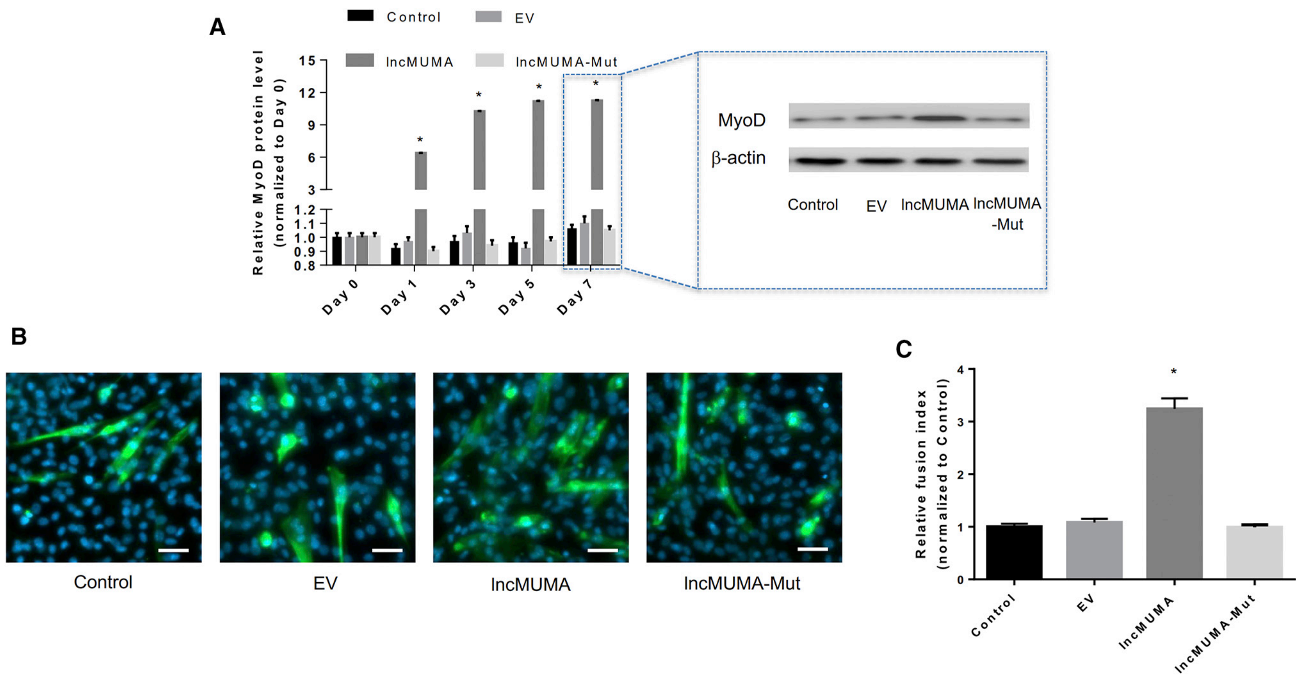

Figure 4. lncMUMA, but Not Mutated lncMUMA, Counteracted the Decreases in MyoD Protein Level and Myotube Formation in C2C12 Cells Stably Overexpressing miR-762 *In Vitro*

(A) (Left) Western blot analysis of MyoD protein in C2C12 cells stably overexpressing miR-762 transfected with empty vector (EV), lncMUMA vector, and mutated lncMUMA vector (lncMUMA-Mut), respectively, on days 0, 1, 3, 5, and 7 of differentiation. (Right) Representative images of MyoD protein in each group on day 7. (B) Representative images of C2C12 cells in each group on day 7 of differentiation. Myosin was labeled with green fluorescence and the nuclei were labeled with DAPI. Scale bars, 50 μm. (C) The fusion index in C2C12 cells in each group on day 7 of differentiation. n = 5 at each time point for each group. β-actin is used as the control for protein. Data are presented as mean ± SEM. *$p < 0.05$ versus the corresponding day 0.

antagomiR-762 (a miR-762 inhibitor) elevated the MyoD protein expression (Figure 3E; Figure S5).

To validate the interaction between miR-762 and MyoD, the luciferase reporter gene containing either wild-type MyoD 3′ UTR-binding site (MyoD 3′ UTR) or mutated MyoD 3′ UTR-binding site for miR-762 (MyoD 3′ UTR-Mut) was constructed (Figure 3F). Luciferase reporter assay revealed that agomiR-762 (agomiR-762 containing wild-type-binding site for MyoD), but not agomiR-762-Mut (agomiR-762 containing mutated binding site for MyoD), could reduce the luciferase activity of MyoD 3′ UTR. Meanwhile, the luciferase activity of MyoD 3′ UTR-Mut was not repressed by agomiR-762 (Figure 3G). Furthermore, the luciferase activity of MyoD 3′ UTR was significantly increased after reducing the endogenous levels of miR-762 by treating C2C12 cells with antagomiR-762 (Figure 3H).

lncMUMA Vector, but Not Mutated lncMUMA Vector, Counteracted the Decreases in Expression of MyoD and Muscle Differentiation in miR-762-Overexpressing C2C12 Cells

To evaluate the effect of interaction between lncMUMA and miR-762 on MyoD protein level and muscle differentiation, lncMUMA plasmid containing either wild-type or mutated binding site of lncMUMA with miR-762 was transfected into the C2C12 cells stably overexpressing miR-762. The expression of MyoD protein and myotube formation stayed at low levels in miR-762-overexpressing C2C12 cells (Figure 4A). Transduction of lncMUMA vector containing wild-type binding site, but not the mutated lncMUMA vector, elevated the MyoD protein level (Figure 4A). Moreover, the abundance of multinuclear myotubes was increased in the wild-type lncMUMA vector group, while the same phenomenon was not observed after mutated lncMUMA vector transduction (Figures 4B and 4C).

Skeletal Muscle-Specific Overexpression of lncMUMA Promoted MyoD, Muscle Mass, Structure, and Function in Muscle-Specific miR-762 Knockin Mice

To facilitate the mechanism study of the anabolic role of lncMUMA *in vivo*, muscle-specific miR-762 knockin mice were generated (Figure 5A). Compared to other tissues and organs, in the skeletal muscles of muscle-specific miR-762 knockin mice miR-762 was highly expressed (n = 10) (Figure S6A). The mice also showed lower muscle mass, lower muscle fiber CSA, and lower MyoD protein level in gastrocnemius muscle compared to the control mice (Figures S6B–S6D). lncMUMA overexpression increased the muscle mass, muscle fiber CSA, and muscle strength in muscle-specific miR-762 mice (n = 10) (Figures 5B–5E). In addition, lncMUMA overexpression elevated the expression level of MyoD protein in gastrocnemius muscle of muscle-specific miR-762 knockin mice (Figure 5F).

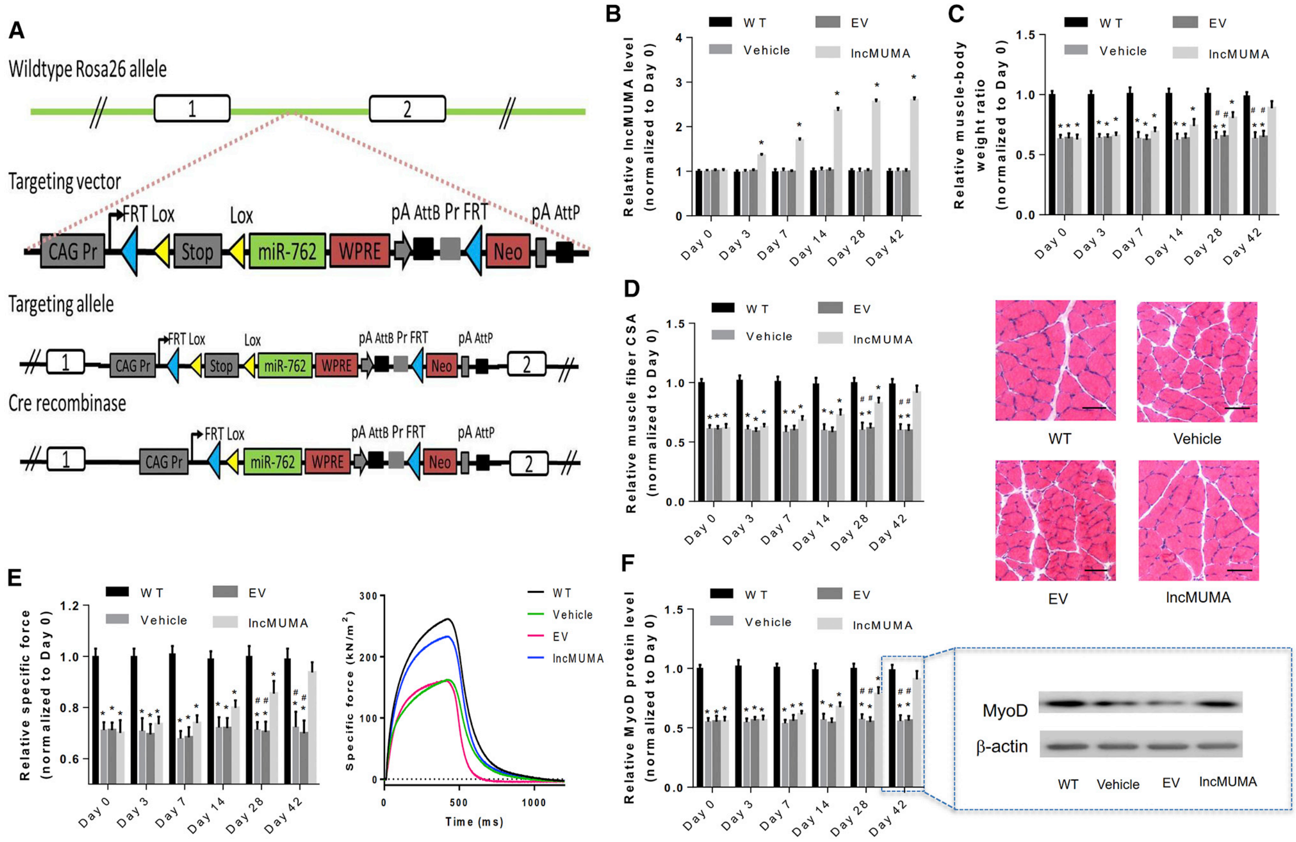

Figure 5. lncMUMA Overexpression Promoted MyoD, Muscle Mass, Structure, and Function in Muscle-Specific miR-762 Knockin Mice

(A) Schematic diagram for the development strategy of miR-762 knockin mice. (B) Real-time PCR analysis of lncMUMA levels in gastrocnemius muscle of wild-type control (WT), vehicle control, empty vector (EV), and lncMUMA vector (lncMUMA) mice, respectively, on days 0, 3, 7, 14, 28, and 42 after vector injection. (C) Gastrocnemius muscle-to-body weight ratio in each group at each time point. (D) (Left) Gastrocnemius muscle fiber CSA in each group at each time point. (Right) Cross-sections from mid-belly gastrocnemius muscle in each group at day 42. Scale bars, 50 μm. (E) (Left) *In situ* muscle function testing of specific force in gastrocnemius muscle of each group at each time point. (Right) Specific force in gastrocnemius muscle of each group at day 42. (F) (Left) Expression level of MyoD protein in each group at each time point. (Right) Representative western blot images of MyoD protein in gastrocnemius muscle of each group at day 42. n = 10 at each time point for these two groups. U6 small nuclear RNA is used as the endogenous control of lncRNA. β-actin is used as the endogenous control for MyoD protein. Data are presented as mean ± SEM. *p < 0.05 versus control, #p < 0.05 versus HLS + lncMUMA.

Enforced lncMUMA Expression Counteracted the Decreases in Muscle Differentiation in MGS C2C12 Myoblasts

To further explore the effect of lncMUMA in counteracting the suppressed muscle differentiation induced by MGS *in vitro*, we infected the C2C12 cells with lncMUMA lentivirus to overexpress the lncMUMA level and then cultured them in an MGS environment. The lncMUMA was stably overexpressed in the lncMUMA group, while lncMUMA expression was found dramatically decreased in the empty vector (EV) group (Figure 6A). There was no significant change in the MyoD protein level in the lncMUMA vector group, while it was significantly decreased in the EV group (Figure 6B). Moreover, much less myotube formed in the EV group compared to that in the lncMUMA group on day 7 of the differentiation in C2C12 cells (Figures 6C and 6D).

Skeletal Muscle-Specific Overexpression of lncMUMA Could Attenuate and Reverse the Decreases of MyoD, Muscle Mass, Structure, and Function following Mechanical Unloading

To evaluate the preventive effect of lncMUMA overexpression on muscle atrophy following mechanical unloading *in vivo*, the adult mice were locally injected with lncMUMA vector in gastrocnemius muscle and then underwent 42-day HLS (n = 10). lncMUMA overexpression attenuated the decreases in muscle histomorphometric (i.e., muscle mass and muscle fiber CSA) and functional (i.e., muscle strength) parameters in HLS mice (Figures S7A–S7D). In addition, the decreased MyoD protein in the gastrocnemius muscle of HLS mice was also successfully attenuated by lncMUMA overexpression (Figure S7E).

To further evaluate the effect of lncMUMA overexpression on reversing established muscle atrophy following mechanical

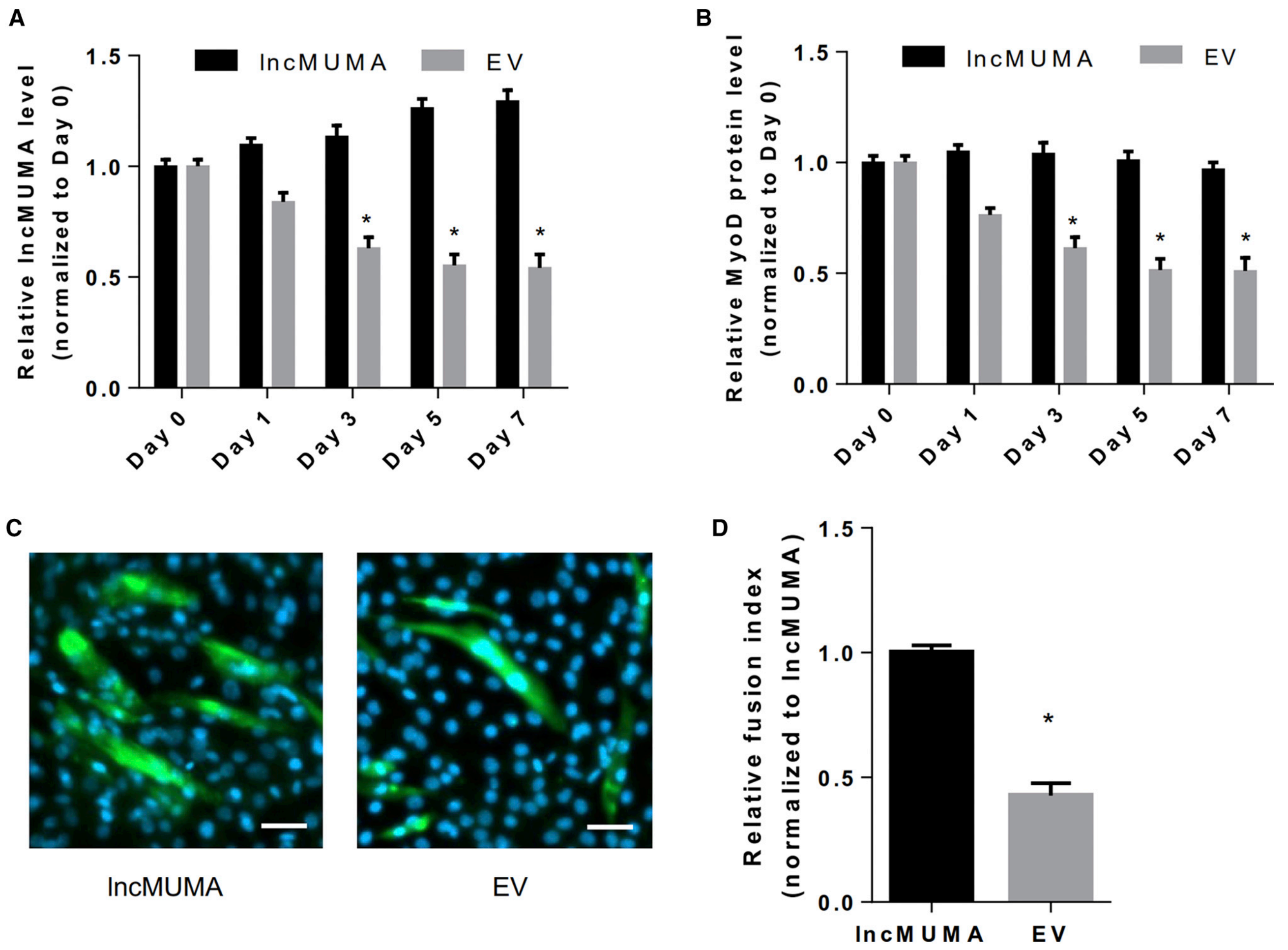

Figure 6. Enforced lncMUMA Expression Could Counteract the Decreases in MyoD Protein Level and Myotube Formation in Microgravity-Simulated C2C12 Myoblasts *In Vitro*

(A) Real-time PCR analysis of lncMUMA levels in C2C12 cells transfected with either lncMUMA overexpression vector (lncMUMA) or empty vector (EV) under microgravity-simulated (MGS) culture environment on days 0, 1, 3, 5, and 7 of differentiation. (B) Western blot analysis of MyoD protein level in C2C12 cells transfected with lncMUMA or EV under MGS culture environment on days 0, 1, 3, 5, and 7 of differentiation. (C) Representative images of C2C12 cells transfected with lncMUMA or EV under MGS culture environment on day 7 of differentiation. Myosin was labeled with green fluorescence and the nuclei were labeled with DAPI. Scale bars, 50 μm. (D) The fusion index in C2C12 cells transfected with lncMUMA or EV under MGS culture environment on day 7 of differentiation. n = 5 at each time point for each group. U6 small nuclear RNA is used as the internal control of lncRNA. β-actin is used as the control for protein. Data are presented as mean ± SEM. *p < 0.05 versus the corresponding day 0.

unloading, the adult mice with established muscle atrophy (on day 14 of HLS) were locally injected with either EV or lncMUMA lentivirus in the gastrocnemius muscle, and they were kept hindlimb suspended until day 42 (n = 10). The decreased muscle mass and muscle fiber CSA started to elevate after lncMUMA overexpression on day 14 of HLS (Figures 7A–7D). Moreover, MyoD protein level and muscle-specific force in the lncMUMA vector group were higher than those in the EV group on day 42 of HLS (Figure 7E).

DISCUSSION

In this study, the lncRNA lncMUMA in mice skeletal muscle that functions as a miR-762 sponge to promote skeletal muscle differentiation has been identified. Enforced expression of lncMUMA prevented the muscle atrophy development and reversed the established muscle atrophy following mechanical unloading in mice. lncMUMA could be a novel therapeutic target for treating muscle atrophy following mechanical unloading.

lncRNAs, including H19,[12,13] linc-MD1,[14] Yam-1,[17] Malat1,[16] and lnc-mg,[15] have been demonstrated to regulate muscle differentiation, while little is known about their function during myogenesis *in vivo*. In this study, the newly identified lncRNA lncMUMA is highly expressed in skeletal muscle and associated with muscle atrophy following mechanical unloading in mice, implying its potential function in regulating muscle differentiation. It is further confirmed that

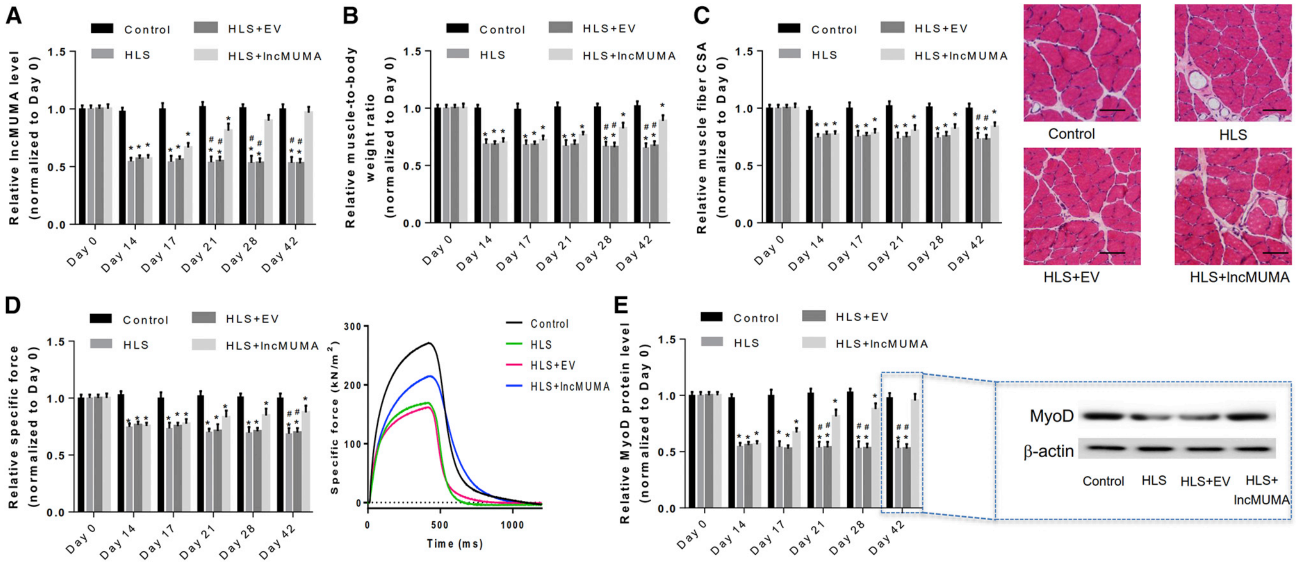

Figure 7. Skeletal Muscle-Specific Overexpression of lncMUMA on Day 14 of HLS Reversed MyoD, Muscle Mass, Structure, and Function in Established Muscle Atrophy following Mechanical Unloading *In Vivo*

(A) Real-time PCR analysis of lncMUMA levels in gastrocnemius muscle of normal control, hindlimb suspension (HLS), HLS + empty vector (EV), and HLS + lncMUMA vector (lncMUMA) mice, respectively, on days 0, 14, 17, 21, 28, and 42 of HLS. (B) Gastrocnemius muscle-to-body weight ratio in each group at each time point. (C) (Left) Gastrocnemius muscle fiber CSA in each group at each time point. (Right) Cross-sections from mid-belly gastrocnemius muscle in each group at day 42 of HLS. Scale bars, 50 μm. (D) (Left) *In situ* muscle function testing of specific force in gastrocnemius muscle of each group at each time point. (Right) Specific force in gastrocnemius muscle of each group at day 42 of HLS. (E) (Left) Expression level of MyoD protein in each group at each time point. (Right) Expression level of MyoD protein in gastrocnemius muscle of each group at day 42 of HLS. Note: the administration of empty or lncMUMA lentivirus was performed on day 14. n = 10 at each time point for each group. U6 small nuclear RNA is used as the internal control of lncRNA. β-actin is used as the control for protein. Data are presented as mean ± SEM. *$p < 0.05$ versus control, $^{\#}p < 0.05$ versus HLS + lncMUMA.

lncMUMA promoted myogenesis *in vitro* and *in vivo*, evidenced by the reduced muscle mass and strength when silencing lncMUMA, which is consistent with the *in vitro* data. Furthermore, enforced lncMUMA expression in skeletal muscle prevents muscle atrophy and even reverses established muscle atrophy following mechanical unloading.

Recently, several studies have reported a regulatory mechanism of lncRNA, that lncRNA may function as ceRNA to sponge miRNA, thereby modulating the derepression of miRNA targets and imposing an additional level of post-transcriptional regulation.[19] In our study, miR-762 is predicted to contain a binding site for lncMUMA by bioinformatics analysis. Pull-down assay and luciferase assay validate the direct binding of the miR-762 response elements on the lncMUMA transcript. Furthermore, the enhanced muscle differentiation in lncMUMA-infected C2C12 cells is attenuated by the wild-type miR-762, but not the mutated miR-762. It has been reported that miR-762 could promote the development of ovarian cancer by suppressing Menin.[20] miR-762 has also been reported to regulate Interferon-induced transmembrane protein 5 (IFITM5), an osteoblast-specific membrane protein.[21] This is the first study to reveal miR-762's role in myogenesis.

MyoD is a key regulator during muscle differentiation.[22] In our study, decreased MyoD protein expression is closely associated with the decreased lncMUMA expression in MGS C2C12 cells and HLS mouse muscle, while the expression level of miR-762 is unchanged. lncMUMA, but not mutated lncMUMA, counteracts the decreases in expression of MyoD and muscle differentiation in miR-762-overexpressed C2C12 cells. Furthermore, lncMUMA overexpression promotes MyoD, muscle mass, structure, and function in muscle-specific miR-762 knockin mice. These data indicate that lncMUMA interacts with miR-762 to post-transcriptionally regulate the MyoD protein.

To date, most studies of lncRNAs in skeletal muscle are investigated in the C2C12 cell line or animals under physiological condition; there's no report to clarify the role of lncRNA in a mechanical unloading-induced muscle atrophy animal model.[15] In this study, we overexpressed lncMUMA level in the skeletal muscle of HLS mice, which is an established animal model of muscle atrophy following mechanical unloading. Skeletal muscle-specific overexpression of lncMUMA before HLS prevents muscle atrophy. Furthermore, it could also reverse established muscle atrophy induced by unloading. The data show the therapeutic potential of lncMUMA in muscle atrophy.

We used mouse α-skeletal actin promoter to construct the lentiviral vectors to achieve skeletal muscle-specific gene expression with high efficiency in our study. The α-skeletal actin plays important roles in skeletal muscle contraction.[23] Its promoter has been used for

skeletal muscle-specific gene delivery due to its high tissue specificity and expression efficiency.[24,25] The lentivirus has been widely used in gene therapy research.[26] It has also been used in the transduction for C2C12 myoblasts[27–30] and skeletal muscle in mice.[31,32] The intramuscular administration of lentiviral particles with α-skeletal actin promoter could generate high-level skeletal muscle-specific gene expression for 2 years.[25] In our study, direct injection of lentiviral particles constructed with mouse α-skeletal actin promoter into the gastrocnemius muscle of mice resulted in the significantly higher expression level of target gene lncMUMA in gastrocnemius muscle from lncRNA lentivirus-treated mice than that in vector control mice.

lncRNAs are appealing therapeutic targets because of the following characteristics: the involvement in disease development; greater tissue, cell, and time specificity than protein-coding genes; and multiple mutually non-exclusive molecular mechanisms of regulating miRNAs or proteins. Plenty of approaches to regulate lncRNAs on a therapeutical level have been studied in a preclinical phase.[33,34] However, there is still no lncRNA-based drug brought into clinical trials to date, especially for skeletal muscle atrophy, due to the difficulty in lncRNA modulation in humans and the potential systematic side effect. Tissue- or organ-specific delivery systems, such as muscle-targeted peptide[35,36] and muscle-targeted aptamer,[37] could be helpful for the lncRNA-based therapy to avoid the potential side effect to other tissues and organs. Another challenge of lncRNA-based therapy is the poor sequence conservation of lncRNA across species. lncRNA conservation includes four dimensions: the sequence, structure, function, and expression from syntenic loci.[38,39] Lack of sequence conservation does not directly imply the lack of function. We have demonstrated that lncMUMA is functionally conserved between mouse and human (Figure S8).

In conclusion, the newly identified lncRNA lncMUMA acts as a miR-762 sponge to regulate MyoD protein, resulting in promoting muscle differentiation. lncMUMA could be a novel anabolic therapeutic agent to reverse established skeletal muscle atrophy following mechanical unloading.

MATERIALS AND METHODS

Animals

6-month-old C57BL/6J mice were used in the study. All the animals were maintained under standard animal housing conditions (12-hr-light and 12-hr-dark cycles and free access to food and water). All the experimental procedures were approved by the Committees of Animal Ethics and Experimental Safety of the Chinese University of Hong Kong.

HLS Procedure

The animals were subjected to HLS for 42 days following established procedure.[40] Briefly, a strip of adhesive tape was applied to the animal's tail, which was suspended by passing the tape through a fishline swivel that was attached to a metal bar on the top of the cage. The forelimbs were allowed to touch the grid floor and the animals could move around the cage for free access to food and water. The suspension height was adjusted to prevent the hindlimbs from touching any supporting surface while maintaining a suspension angle of approximately 30°. The distal tip of the tail was examined to ensure that the procedure did not occlude blood flow to the tail.

Cell Culture

The C2C12 mouse myoblast cell line was supplied by American Type Culture Collection (ATCC). Cells were cultured at subconfluent densities in growth medium made up of DMEM supplemented with 10% heat-inactivated fetal calf serum (FCS) and 1% penicillin-streptomycin. C2C12 myoblast cells were differentiated into myocytes or myotubes in differentiation medium, consisting of DMEM containing 2% heat-inactivated horse serum and 1% penicillin-streptomycin. All these cells were maintained in a humidified atmosphere containing 5% CO_2 at 37°C.[41]

Random Positioning Machine

For the cell culture under MGS environment, a random positioning machine (RPM) (Dutch Space, Leiden, the Netherlands) was used. The RPM is in essence a 3-dimensional clinostat in which the gravity vector is continually reoriented, allowing the simulation of microgravity conditions. The cells were plated into a T-25 flask or 35-mm cover-glass-bottom dish. The dish was sealed with gas-permeable parafilm to avoid the leakage of the medium, and the dishes were fixed near the center of 2 frames that simultaneously rotated independently of each other. The entire system was placed in an incubator set at 37°C. Control samples (1 g) were placed in the same incubator and near the RPM. Microgravity simulation lasted from 1 to 7 days. The medium was replaced with fresh medium every 72 hr.[42]

Immunocytochemistry Evaluation and Fusion Index

Cells were fixed using 4% formaldehyde in PBS for 15 min and then washed with PBS twice for 5 min. Fixed cells were permeabilized and blocked with 1% BSA (Bioshop) in PBST (PBS with 0.25% Triton X-100) for 30 min at room temperature. The cells were washed once with PBST and incubated in anti-myosin heavy chain (MyHC, 1:100; R&D Systems) supernatant for 1 hr at room temperature. The cells were washed with PBST three times and incubated with AlexFluor488 secondary antibody (1:250; Cell Signaling Technology) in blocking solution for 1 hr in the dark at room temperature. Following extensive washes with PBS, cells were mounted in mounting medium with DAPI (Merck KGaA) and imaged using the Zeiss Observer Z1 microscope. The fusion index was determined by quantifying the number of nuclei within multinucleated MyHC-expressing myotubes divided by total nuclei using ImageJ software (NIH).

Isolation of Total RNA and Real-Time PCR Analysis

The total RNA from cell line and tissue samples was isolated by Trizol reagent (Invitrogen), following the manufacturer's instructions. cDNA synthesis for mRNA and lncRNA detection was carried out using SuperScript III first strand synthesis system for RT-PCR (Invitrogen). The Fast start Universal SYBR Green Master (Roche) was applied for the qRT-PCR. Quantification of amplicons was

done using ABI 7300 software (Applied Biosystems). GAPDH and U6 were used as endogenous controls for normalization. All the primer sequences are listed in Table S2. The relative fold changes of candidate genes were analyzed by using the $2^{-\Delta\Delta CT}$ method.[41]

Microarray Analysis

150 ng total RNA was used to generate amplified and biotinylated sense-strand cDNA with the GeneChip WT PLUS Reagent Kit (Affymetrix), according to the manufacturer's instructions. cDNA was hybridized to GeneChip Mouse Transcriptome Array 1.0 (Affymetrix, Thermo Fisher Scientific) for 16 hr in a 45°C incubator, rotated at 60 rpm. After hybridization, the microarrays were washed, and then they were stained using the Fluidics Station 450 followed by scanning with the Affymetrix GeneChip Scanner 3000 7G. The data were analyzed in Expression Console software from Affymetrix using default analysis settings. Differentially expressed lncRNAs were identified when the paired t test p value was <0.05 and the fold change was greater than 2. The sample size was three mice per group.[43]

Lentiviral Vector Construction and Lentivirus Production

For construction of the lncMUMA overexpression lentiviral vector, mouse α-skeletal actin (MSA) promoter (2.0 kb) and full-length lncMUMA were subcloned into the lentiviral GV112 vector, which was provided by Shanghai Genechem (Shanghai, China), according to the manufacturer's instructions.[44] For the lncMUMA-KD lentiviral vector, shRNA targeting lncMUMA or a negative control scramble sequence was subcloned into the GV112 vector, respectively. Three shRNA sequences were designed by Shanghai Genechem: shlncMUMA-1, 5′-GACAGTTGTATACCTTTCTCTTG-3′; shlncMUMA-2, 5′-ACCATGTATGGGGTAGACTTTTG-3′; and shlncMUMA-3, 5′-GCCATGTATACACTGTGTAAATG-3′.

For the production of lentivirus, the expression vectors were co-transfected with packaging plasmid pHelper 1.0 vector (Shanghai Genechem) and envelope plasmid pHelper 2.0 vector (Shanghai Genechem) into 293T cells using TransIT-LT1 (Mirus Bio). The supernatant was collected 48 and 72 hr post-transfection, concentrated by ultracentrifugation at 25,000 rpm for 90 min, and resuspended in an appropriate volume of OptiMEM (Gibco). The infectious particle titer (IU/mL) was determined by real-time qPCR.[45]

Cell Transduction

Based on a previously reported protocol with modification,[30] C2C12 mouse myoblasts were seeded in 6-well plates and cultured until 60% confluent. Medium was then removed and 1.5×10^8 IU viral particles were added together with 8 μg/mL hexadimethrine bromide (Sigma-Aldrich). The medium volume was made up to 500 μL with DMEM and then incubated at 37°C and 5% CO_2 for 8 hr, at which time the DMEM plus viral particles was changed to DMEM with 10% FCS and the cells were cultured for 1–7 days.

Bioinformatics Analysis for Targeted Gene Prediction

The corresponding targeted genes for lncMUMA and miR-762 were predicted using RNAhybrid 2.12 (https://bibiserv.cebitec.uni-bielefeld.de/rnahybrid/). RNAhybrid is a tool used for predicting biological targets of certain RNA by searching for the presence of conserved sites that match the seed region of an RNA.[46]

Luciferase Reporter Assays

Following a previously established procedure, the putative sequences of the binding site in miR-762 and the mutated sequences were cloned into a pmirGlO Dual-luciferase miRNA Target Expression Vector (Promega, Madison, WI, USA) to form the reporter vector.[41] The reporter vector was co-transfected with lncMUMA-WT or lncMUMA-Mut into C2C12 cells by using Lipofectamine 2000 (Invitrogen). After 48 hr, luciferase assay was performed using a Dual-Luciferase Reporter Assay System (Promega) according to the manufacturer's protocol.

Biotin-Labeled lncMUMA Pulled down

Biotin-labeled lncMUMA was synthesized by Sangon Biotech (Shanghai, China). The C2C12 cells were transfected with miR-762 by using Lipofectamine 2000 (Invitrogen). Different doses of biotin-labeled lncMUMA (0.5, 5, and 50 mM) were incubated with cytoplasmic lysates from miR-762-transfected C2C12 cells for 30 min at room temperature, and complexes were isolated with streptavidin-coated magnetic Dynabeads (Dynal). After wash steps, the captured RNA was purified and analyzed with real-time PCR.[47]

Western Blotting

According to previously established procedures, cells and muscle samples were harvested, washed with 1× PBS, and lysed in NP40 lysis buffer (50 mM Tris-HCl, 150 mM NaCl, 0.1% NP-40, 5 mM EDTA, and 10% glycerol) with protease inhibitor cocktail (Sigma).[41] Proteins were separated in SDS-PAGE, transferred, and immunoblotted with various antibodies. The antibodies used were anti-MyoD (1:1,000; Invitrogen) and anti-β-actin (dilution 1:3,000; Santa Cruz Biotechnology).

Generation of Muscle-Specific miR-762 Knockin Mice

For the generation of ROSA26-PCAG-STOP^{flox}-miR-762-EGFP mice, targeting vector was constructed by inserting a STOP^{flox}-miR-762-EGFP in the ROSA26 allele and then electroporating into embryonic stem cells from C57BL/6J mice. Then, these mice were intercrossed with Tg(ACTA1-cre/Esr1*)2Kesr/J (ACTA1-Cre) mice (The Jackson Laboratory), and the offspring were intraperitoneally administered with tamoxifen (500 mg in 200 μL PBS/day) for 5 days at the age of 2 months old to induce muscle-specific miR-762 knockin.[24] The ACTA1 gene encodes skeletal muscle α-actin, which is the predominant actin isoform in the sarcomeric thin filaments of adult skeletal muscle.[48] Thus, miR-762 was overexpressed in skeletal muscle under the control of the ACTA1 gene in the muscle-specific miR-762 knockin mice.

In Vivo Lentiviral Particle Administration

Following established protocols with minor modifications,[49] the animals were anesthetized with an isophorone gas inhale system, and 15 μL viral preparation with a titer of 10.0×10^8 IU/mL was injected into the mid-portion of gastrocnemius muscles by using a Hamilton syringe (Sigma-Aldrich) with a 33G needle. The mice were sacrificed

after 42-day HLS. The specific force generated by gastrocnemius muscle was evaluated by *in situ* muscle functional testing before sacrifice. The gastrocnemius muscles were collected and weighed. The muscles were cryosectioned for histological staining. The mean muscle fiber CSA was determined. lncMUMA level and mRNA were assessed by real-time PCR. The protein of MyoD was evaluated by western blot analysis.

In Situ Muscle Functional Testing

In situ muscle functional testing was performed following the established protocol.[50] The animals were anesthetized with an isophorone gas inhale system and then placed on a 37°C heated platform. The gastrocnemius muscle was exposed, and the distal tendon was attached to the lever arm of a position feedback motor. The knee joint was immobilized by clamping it to the platform (In Vivo Muscle Test system 1300A, Aurora Scientific). A needle electrode was inserted through the skin and positioned on the peroneal nerve to stimulate contraction of the gastrocnemius muscle. The peak isometric force at 100 Hz for 400 ms was recorded and calculated with the ASI Dynamic Muscle Control Software (DMC version [v.]5.1 beta, Aurora Scientific). Specific force was expressed as peak tetanic force normalized to the muscle physical CSA.

Histology Evaluation

The dissected gastrocnemius muscles were snap frozen in liquid nitrogen-cooled isopentane and then embedded in optimal cutting temperature (OCT) medium. Serial cross-sections (6-μm thickness) were cut from the mid-belly of the muscles on a cryostat at −20°C for histological staining. H&E staining was performed to examine the general morphology and to determine the CSA of the muscle fiber. Slides were visualized with an Axio Observer Z1 microscope (Carl Zeiss). The muscle area and the number of fibers were determined using ImageJ software (NIH). The mean muscle fiber CSA was obtained by dividing the area of over 300 muscle fibers in 6 different locations in one slide by the fiber number.[51]

Statistical Analysis

Data are presented as mean ± SEM. Each separate sample was analyzed in triplicate to yield an average value, and "n" is the number of samples in each group (n = 3 for microarray analysis, n = 5 for *in vitro* evaluation, and n = 10 for *in vivo* evaluation). GraphPad Prism v.6.0 was used for statistical analysis of the data. All results between groups were analyzed by applying the one-way ANOVA[52] and Student's t test. A difference with $p < 0.05$ was regarded as statistical significance.

SUPPLEMENTAL INFORMATION

Supplemental Information includes eight figures and two tables and can be found with this article online at https://doi.org/10.1016/j.ymthe.2018.09.014.

AUTHOR CONTRIBUTIONS

B.-T.Z., G.Z., and A.L. supervised the whole project. Z.-K.Z. performed the animal study and wrote the manuscript. J. Li performed the *in vitro* study and interpreted the data. D.G. and C.L. performed the microarray data analysis and bioinformatics analysis. Z.Z. and J. Liu provided the technical support and their professional expertise.

CONFLICTS OF INTEREST

The authors have no conflicts of interest.

ACKNOWLEDGMENTS

This study was supported by the Hong Kong General Research Fund (14112915), the direct grant of The Chinese University of Hong Kong (4054220 and 4054138), the Faculty Research Grant of Hong Kong Baptist University (FRG2/15-16/038), and the Natural Science Foundation Council of China (31501080).

REFERENCES

1. Lambertz, D., Pérot, C., Kaspranski, R., and Goubel, F. (2001). Effects of long-term spaceflight on mechanical properties of muscles in humans. J. Appl. Physiol. (1985) *90*, 179–188.
2. Fitts, R.H., Trappe, S.W., Costill, D.L., Gallagher, P.M., Creer, A.C., Colloton, P.A., Peters, J.R., Romatowski, J.G., Bain, J.L., and Riley, D.A. (2010). Prolonged space flight-induced alterations in the structure and function of human skeletal muscle fibres. J. Physiol. *588*, 3567–3592.
3. Torgan, C.E., Burge, S.S., Collinsworth, A.M., Truskey, G.A., and Kraus, W.E. (2000). Differentiation of mammalian skeletal muscle cells cultured on microcarrier beads in a rotating cell culture system. Med. Biol. Eng. Comput. *38*, 583–590.
4. Stein, T.P., Larina, I.M., Leskiv, M.J., and Schluter, M.D. (2000). [Protein turnover during and after extended space flight]. Aviakosm. Ekolog. Med. *34*, 12–16.
5. Trappe, S., Costill, D., Gallagher, P., Creer, A., Peters, J.R., Evans, H., Riley, D.A., and Fitts, R.H. (2009). Exercise in space: human skeletal muscle after 6 months aboard the International Space Station. J. Appl. Physiol. (1985) *106*, 1159–1168.
6. Drummond, M.J., and Rasmussen, B.B. (2008). Leucine-enriched nutrients and the regulation of mammalian target of rapamycin signalling and human skeletal muscle protein synthesis. Curr. Opin. Clin. Nutr. Metab. Care *11*, 222–226.
7. Zwart, S.R., Davis-Street, J.E., Paddon-Jones, D., Ferrando, A.A., Wolfe, R.R., and Smith, S.M. (2005). Amino acid supplementation alters bone metabolism during simulated weightlessness. J. Appl. Physiol. (1985) *99*, 134–140.
8. Alzghoul, M.B., Gerrard, D., Watkins, B.A., and Hannon, K. (2004). Ectopic expression of IGF-I and Shh by skeletal muscle inhibits disuse-mediated skeletal muscle atrophy and bone osteopenia in vivo. FASEB J. *18*, 221–223.
9. Gregory, C.M., Vandenborne, K., Huang, H.F., Ottenweller, J.E., and Dudley, G.A. (2003). Effects of testosterone replacement therapy on skeletal muscle after spinal cord injury. Spinal Cord *41*, 23–28.
10. Finkle, W.D., Greenland, S., Ridgeway, G.K., Adams, J.L., Frasco, M.A., Cook, M.B., Fraumeni, J.F., Jr., and Hoover, R.N. (2014). Increased risk of non-fatal myocardial infarction following testosterone therapy prescription in men. PLoS ONE *9*, e85805.
11. Lu, L., Zhu, G., Zhang, C., Deng, Q., Katsaros, D., Mayne, S.T., Risch, H.A., Mu, L., Canuto, E.M., Gregori, G., et al. (2012). Association of large noncoding RNA HOTAIR expression and its downstream intergenic CpG island methylation with survival in breast cancer. Breast Cancer Res. Treat. *136*, 875–883.
12. Borensztein, M., Monnier, P., Court, F., Louault, Y., Ripoche, M.A., Tiret, L., Yao, Z., Tapscott, S.J., Forné, T., Montarras, D., and Dandolo, L. (2013). Myod and H19-Igf2 locus interactions are required for diaphragm formation in the mouse. Development *140*, 1231–1239.
13. Kallen, A.N., Zhou, X.B., Xu, J., Qiao, C., Ma, J., Yan, L., Lu, L., Liu, C., Yi, J.S., Zhang, H., et al. (2013). The imprinted H19 lncRNA antagonizes let-7 microRNAs. Mol. Cell *52*, 101–112.
14. Cesana, M., Cacchiarelli, D., Legnini, I., Santini, T., Sthandier, O., Chinappi, M., Tramontano, A., and Bozzoni, I. (2011). A long noncoding RNA controls muscle differentiation by functioning as a competing endogenous RNA. Cell *147*, 358–369.

15. Zhu, M., Liu, J., Xiao, J., Yang, L., Cai, M., Shen, H., Chen, X., Ma, Y., Hu, S., Wang, Z., et al. (2017). Lnc-mg is a long non-coding RNA that promotes myogenesis. Nat. Commun. *8*, 14718.

16. Watts, R., Johnsen, V.L., Shearer, J., and Hittel, D.S. (2013). Myostatin-induced inhibition of the long noncoding RNA Malat1 is associated with decreased myogenesis. Am. J. Physiol. Cell Physiol. *304*, C995–C1001.

17. Lu, L., Sun, K., Chen, X., Zhao, Y., Wang, L., Zhou, L., Sun, H., and Wang, H. (2013). Genome-wide survey by ChIP-seq reveals YY1 regulation of lincRNAs in skeletal myogenesis. EMBO J. *32*, 2575–2588.

18. Rehmsmeier, M., Steffen, P., Hochsmann, M., and Giegerich, R. (2004). Fast and effective prediction of microRNA/target duplexes. RNA *10*, 1507–1517.

19. Su, Z., Zhi, X., Zhang, Q., Yang, L., Xu, H., and Xu, Z. (2016). LncRNA H19 functions as a competing endogenous RNA to regulate AQP3 expression by sponging miR-874 in the intestinal barrier. FEBS Lett. *590*, 1354–1364.

20. Hou, R., Yang, Z., Wang, S., Chu, D., Liu, Q., Liu, J., and Jiang, L. (2017). miR-762 can negatively regulate menin in ovarian cancer. OncoTargets Ther. *10*, 2127–2137.

21. Mo, X., Lu, Y., and Han, J. (2014). Effects of targeted modulation of miR-762 on expression of the IFITM5 gene in Saos-2 cells. Intractable Rare Dis. Res. *3*, 12–18.

22. Endo, T. (2015). Molecular mechanisms of skeletal muscle development, regeneration, and osteogenic conversion. Bone *80*, 2–13.

23. Sparrow, J.C., Nowak, K.J., Durling, H.J., Beggs, A.H., Wallgren-Pettersson, C., Romero, N., Nonaka, I., and Laing, N.G. (2003). Muscle disease caused by mutations in the skeletal muscle alpha-actin gene (ACTA1). Neuromuscul. Disord. *13*, 519–531.

24. McCarthy, J.J., Srikuea, R., Kirby, T.J., Peterson, C.A., and Esser, K.A. (2012). Inducible Cre transgenic mouse strain for skeletal muscle-specific gene targeting. Skelet. Muscle *2*, 8.

25. Kimura, E., Li, S., Gregorevic, P., Fall, B.M., and Chamberlain, J.S. (2010). Dystrophin delivery to muscles of mdx mice using lentiviral vectors leads to myogenic progenitor targeting and stable gene expression. Mol. Ther. *18*, 206–213.

26. Sharon, D., and Kamen, A. (2018). Advancements in the design and scalable production of viral gene transfer vectors. Biotechnol. Bioeng. *115*, 25–40.

27. Papini, N., Anastasia, L., Tringali, C., Dileo, L., Carubelli, I., Sampaolesi, M., Monti, E., Tettamanti, G., and Venerando, B. (2012). MmNEU3 sialidase over-expression in C2C12 myoblasts delays differentiation and induces hypertrophic myotube formation. J. Cell. Biochem. *113*, 2967–2978.

28. Milazzotto, M.P., Goissis, M.D., Feitosa, W.B., Martins, L.F., Strauss, B.E., Bajgelman, M.C., Assumpção, M.E., and Visintin, J.A. (2010). Myostatin gene knockdown through lentiviral-mediated delivery of shRNA for in vitro production of transgenic bovine embryos. Zygote *18*, 339–344.

29. Roudbari, Z., Nassiri, M.R., Tahmoorespur, M., and Haddad-Mashadrizeh, A. (2016). Lentiviral mediated overexpression of Insulin like Growth Factor-1 in mouse myoblast. Cell. Mol. Biol. *62*, 111–115.

30. Jackson, M.F., Hoversten, K.E., Powers, J.M., Trobridge, G.D., and Rodgers, B.D. (2013). Genetic manipulation of myoblasts and a novel primary myosatellite cell culture system: comparing and optimizing approaches. FEBS J. *280*, 827–839.

31. Eleftheriadou, I., Manolaras, I., Irvine, E.E., Dieringer, M., Trabalza, A., and Mazarakis, N.D. (2016). αCAR IGF-1 vector targeting of motor neurons ameliorates disease progression in ALS mice. Ann. Clin. Transl. Neurol. *3*, 752–768.

32. Van Pham, P., Vu, N.B., Nguyen, H.T., Dao, T.T., Le, H.T., Phi, L.T., Nguyen, O.T., and Phan, N.K. (2017). ETV-2 activated proliferation of endothelial cells and attenuated acute hindlimb ischemia in mice. In Vitro Cell. Dev. Biol. Anim. *53*, 616–625.

33. Meng, L., Ward, A.J., Chun, S., Bennett, C.F., Beaudet, A.L., and Rigo, F. (2015). Towards a therapy for Angelman syndrome by targeting a long non-coding RNA. Nature *518*, 409–412.

34. Piccoli, M.T., Gupta, S.K., Viereck, J., Foinquinos, A., Samolovac, S., Kramer, F.L., Garg, A., Remke, J., Zimmer, K., Batkai, S., and Thum, T. (2017). Inhibition of the Cardiac Fibroblast-Enriched lncRNA *Meg3* Prevents Cardiac Fibrosis and Diastolic Dysfunction. Circ. Res. *121*, 575–583.

35. Seow, Y., Yin, H., and Wood, M.J. (2010). Identification of a novel muscle targeting peptide in mdx mice. Peptides *31*, 1873–1877.

36. Gao, X., Zhao, J., Han, G., Zhang, Y., Dong, X., Cao, L., Wang, Q., Moulton, H.M., and Yin, H. (2014). Effective dystrophin restoration by a novel muscle-homing peptide--morpholino conjugate in dystrophin-deficient mdx mice. Mol. Ther. *22*, 1333–1341.

37. Philippou, S., Mastroyiannopoulos, N.P., Makrides, N., Lederer, C.W., Kleanthous, M., and Phylactou, L.A. (2018). Selection and Identification of Skeletal-Muscle-Targeted RNA Aptamers. Mol. Ther. Nucleic Acids *10*, 199–214.

38. Johnsson, P., Lipovich, L., Grandér, D., and Morris, K.V. (2014). Evolutionary conservation of long non-coding RNAs; sequence, structure, function. Biochim. Biophys. Acta *1840*, 1063–1071.

39. Diederichs, S. (2014). The four dimensions of noncoding RNA conservation. Trends Genet. *30*, 121–123.

40. Zhang, B.T., Yeung, S.S., Liu, Y., Wang, H.H., Wan, Y.M., Ling, S.K., Zhang, H.Y., Li, Y.H., and Yeung, E.W. (2010). The effects of low frequency electrical stimulation on satellite cell activity in rat skeletal muscle during hindlimb suspension. BMC Cell Biol. *11*, 87.

41. Zhang, Z.K., Li, J., Liu, J., Guo, B., Leung, A., Zhang, G., and Zhang, B.T. (2016). Icaritin requires Phosphatidylinositol 3 kinase (PI3K)/Akt signaling to counteract skeletal muscle atrophy following mechanical unloading. Sci. Rep. *6*, 20300.

42. Benavides Damm, T., Franco-Obregón, A., and Egli, M. (2013). Gravitational force modulates G2/M phase exit in mechanically unloaded myoblasts. Cell Cycle *12*, 3001–3012.

43. Wang, M., Liu, W., Jiao, J., Li, J., Wang, C., and Zhang, L. (2017). Expression Profiling of mRNAs and Long Non-Coding RNAs in Aged Mouse Olfactory Bulb. Sci. Rep. *7*, 2079.

44. Ou, L., Przybilla, M.J., Koniar, B.L., and Whitley, C.B. (2016). Elements of lentiviral vector design toward gene therapy for treating mucopolysaccharidosis I. Mol. Genet. Metab. Rep. *8*, 87–93.

45. Benchaouir, R., Meregalli, M., Farini, A., D'Antona, G., Belicchi, M., Goyenvalle, A., Battistelli, M., Bresolin, N., Bottinelli, R., Garcia, L., and Torrente, Y. (2007). Restoration of human dystrophin following transplantation of exon-skipping-engineered DMD patient stem cells into dystrophic mice. Cell Stem Cell *1*, 646–657.

46. Wang, D., Zhou, J., Liu, X., Lu, D., Shen, C., Du, Y., Wei, F.Z., Song, B., Lu, X., Yu, Y., et al. (2013). Methylation of SUV39H1 by SET7/9 results in heterochromatin relaxation and genome instability. Proc. Natl. Acad. Sci. USA *110*, 5516–5521.

47. Li, S., Kimura, E., Fall, B.M., Reyes, M., Angello, J.C., Welikson, R., Hauschka, S.D., and Chamberlain, J.S. (2005). Stable transduction of myogenic cells with lentiviral vectors expressing a minidystrophin. Gene Ther. *12*, 1099–1108.

48. Laing, N.G., Dye, D.E., Wallgren-Pettersson, C., Richard, G., Monnier, N., Lillis, S., Winder, T.L., Lochmüller, H., Graziano, C., Mitrani-Rosenbaum, S., et al. (2009). Mutations and polymorphisms of the skeletal muscle alpha-actin gene (ACTA1). Hum. Mutat. *30*, 1267–1277.

49. Matsuba, Y., Goto, K., Morioka, S., Naito, T., Akema, T., Hashimoto, N., Sugiura, T., Ohira, Y., Beppu, M., and Yoshioka, T. (2009). Gravitational unloading inhibits the regenerative potential of atrophied soleus muscle in mice. Acta Physiol. (Oxf.) *196*, 329–339.

50. Ashton-Miller, J.A., He, Y., Kadhiresan, V.A., McCubbrey, D.A., and Faulkner, J.A. (1992). An apparatus to measure in vivo biomechanical behavior of dorsi- and plantarflexors of mouse ankle. J. Appl. Physiol. (1985) *72*, 1205–1211.

51. Zhang, B.T., Yeung, S.S., Cheung, K.K., Chai, Z.Y., and Yeung, E.W. (2014). Adaptive responses of TRPC1 and TRPC3 during skeletal muscle atrophy and regrowth. Muscle Nerve *49*, 691–699.

52. Iliev, R., Kleinova, R., Juracek, J., Dolezel, J., Ozanova, Z., Fedorko, M., Pacik, D., Svoboda, M., Stanik, M., and Slaby, O. (2016). Overexpression of long non-coding RNA TUG1 predicts poor prognosis and promotes cancer cell proliferation and migration in high-grade muscle-invasive bladder cancer. Tumour Biol. *37*, 13385–13390.

YMTHE, Volume *26*

Supplemental Information

Long Noncoding RNA lncMUMA Reverses Established Skeletal Muscle Atrophy following Mechanical Unloading

Zong-Kang Zhang, Jie Li, Daogang Guan, Chao Liang, Zhenjian Zhuo, Jin Liu, Aiping Lu, Ge Zhang, and Bao-Ting Zhang

>AK014246

TTTGTACATGACAGTTGTATACCTTTCTCTTGTATCTGACACAAAACCTATGATAAAT
ACTCAAACAATCCCGACTGGATCGAGGAACCATGTATGGGGTAGACTTTTGTATTAA
GAGCCAAGAAAGGGAGCTTACGAATCATGTTTCCTACGTTTTTATACTGTTTGTTTTA
AACGGCAGCCCTGCCCAGTGGGTTCAGCTTTTCTAGGAAGTGAATGTCAGTACTGG
TGTTTTCTATAGGAATGGAGAAGCCATGTATACACTGTGTAAATGCTCATGTGAGAA
TGACCTAGCGGCACAATCTGACTTGCCTTGGCCTCTGGCCTTCCGGTTACTGTTTTT
GGCAGCTCTCTCTACCTTCCTCTATCCTCAAACCTTGTGCCTGTAGCTTTGTACTTC
AGCTCCCAGGGATAGGAACAGACCTAGTGAACATTCCACGGTGCCTGATCTCGCTG
GCAACTGAGTCCAGCTAGGGCCTGACCCAGCGTCAGTCTCCAAAGCTCTGCTTCC
GGATTCCAAACACTGGCGTGAGGGGCAGTAGTCAGCACTTCTAGATCACCATCTAG
TGAGTCGCTGGTGTAGAGTGAACTTTTACTGCACACTAAGGGCTCACAATTAAAATA
AACCAGAATAGCTTTTTGCTCATGGTAACCAAGTTCAGTGTCTGTGGGGCCCACAG
CCTGGCAGGTCTGGCCCAGCTCTCTGTCACCTGCTGTGGGAGATGGACCGTTTGA
CCTCTCTGGAGGCTGAGACCCCATACCTGCTGCTCAGTGCTGGTAATCAGCCCTCC
CCAGAGTGCGTGCCGGCCAGAGGGCTCCACCCACAGTCCCTGGTCATGTACGCAC
ATCACACTCTTCCTGCCTCTGCACTATGAAAACCATACTGGGAGTTTTAGAAGTGCT
CACTCTTGTACGAGTGTCTGCGGACATGTGTAAAATAAACGTTAAACTCTGCTTCG

Figure S1 Sequence of lncMUMA

Table S1 Top 50 down-regulated and top 50 up-regulated lncRNAs in HLS muscle tissues

No.	Gene Name	Fold Change	No.	Gene Name	Fold Change	No.	Gene Name	Fold Change	No.	Gene Name	Fold Change
Top 50 Down-regulated lncRNAs						Top 50 Up-regulated lncRNAs					
1	AK014246 (lncMUMA)	21.7411	26	AK019096	3.950669	1	AK133680	18.32983	26	AK142736	3.894358
2	LOC100046151	12.95444	27	Gm2710	3.931119	2	AK006720	16.05375	27	AK050117	3.80183
3	1600014K23Rik	10.90429	28	Gm8709	3.917914	3	AK010427	14.75385	28	AK080530	3.772047
4	LOC101056557	9.30606	29	AK164498	3.917582	4	AK008861	10.43807	29	AK048321	3.739837
5	AK019626	8.573749	30	AK004808	3.782735	5	AK037838	8.354839	30	AK018045	3.690979
6	AK040479	8.236479	31	AK090094	3.758475	6	AK050666	8.166809	31	AK005231	3.687683
7	AK088055	7.813218	32	AK017133	3.742424	7	AK158068	6.773596	32	AK041461	3.684211
8	AK140828	6.713292	33	AK137267	3.695415	8	AK172445	6.474126	33	AK002372	3.6197
9	AK089627	5.625397	34	AK008597	3.643666	9	AK003280	5.942007	34	AK011222	3.550058
10	LOC636901	5.614903	35	AK038180	3.630421	10	AK172311	5.595007	35	AK012252	3.533306
11	Gm15453	5.529144	36	Gm5396	3.613182	11	AK165502	5.37358	36	AK003389	3.495472
12	Gm10052	5.313084	37	AK141617	3.575356	12	AK003454	5.304729	37	AK003573	3.388748
13	AK137307	5.281147	38	AK156638	3.568095	13	AK013490	5.049814	38	AK172322	3.379735
14	0610009O20Rik	5.116497	39	AK132982	3.560905	14	AK142700	4.890915	39	AK135737	3.37448
15	LOC100045968	4.969623	40	Gm6498	3.549732	15	AK172626	4.823841	40	AK162281	3.347527
16	AK032367	4.934769	41	AK043296	3.522517	16	AK004150	4.691858	41	AK013679	3.311203
17	AK138117	4.755483	42	Gm5506	3.476566	17	AK002796	4.390714	42	AK087626	3.261159
18	AK075592	4.485645	43	Gm13277	3.436207	18	AK038876	4.381345	43	AK136079	3.226031
19	AK033909	4.429835	44	AK039307	3.397279	19	AK002224	4.353103	44	AK136295	3.191652
20	AK087321	4.341787	45	AK036353	3.363755	20	AK011347	4.26089	45	AK021173	3.167185
21	AK146676	4.259343	46	AK030937	3.34493	21	AK014372	4.121564	46	AK046094	3.165406
22	AK028893	4.205192	47	AK050744	3.313224	22	AK003535	4.115442	47	AK131727	3.142544
23	AK088710	4.180129	48	AK156875	3.288136	23	AK014142	4.054426	48	AK144859	3.138059
24	AK157223	4.115882	49	Gm5331	3.262516	24	AK005641	4.001631	49	AK013681	3.101982
25	AK034076	4.070582	50	AK081791	3.238565	25	AK084371	3.95384	50	AK016405	3.094581

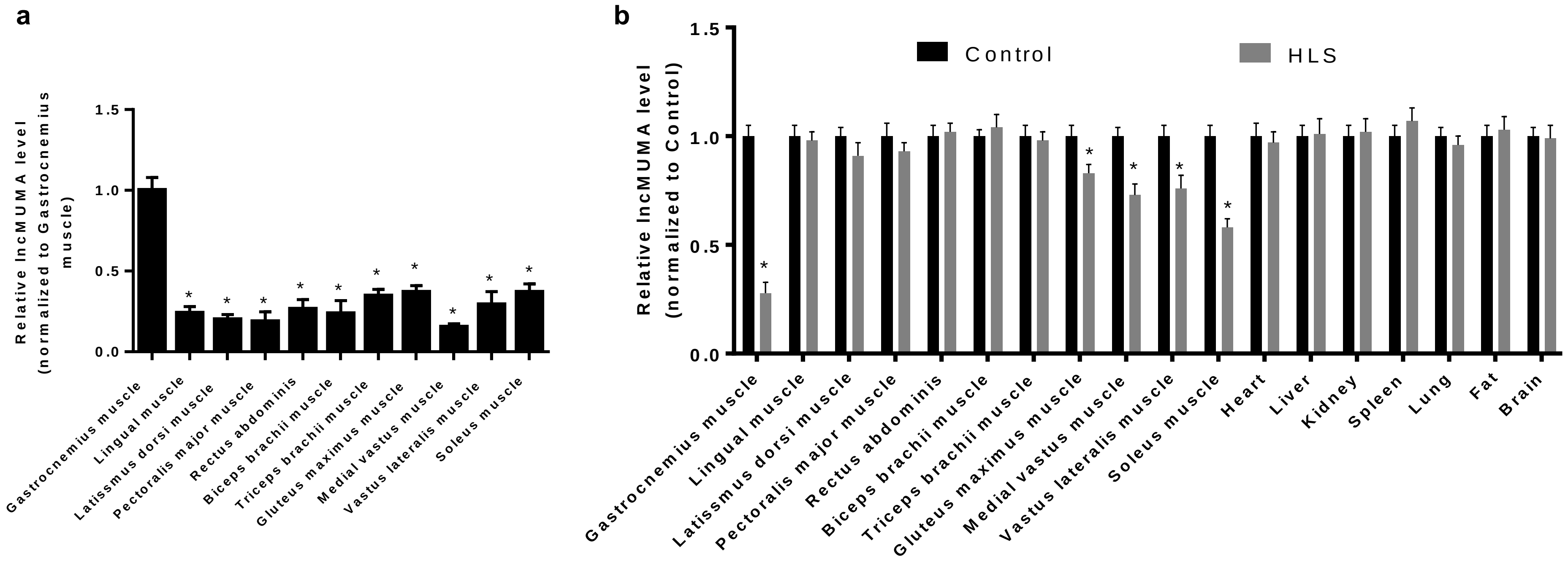

Figure S2 LncMUMA was highly expressed in gastrocnemius muscle and didn't significantly alter in forelimb muscles and other tissue/organs after HLS. (**a**) Real-time PCR analysis of lncMUMA levels in different skeletal muscles of mice. * *P*<0.05 vs. Gastrocnemius muscle. (**b**) Real-time PCR analysis of lncMUMA levels in different skeletal muscles and other tissue/organs of mice with or without HLS treatment. n=10. Data are presented as mean ± SEM. * *P*<0.05 vs. Control.

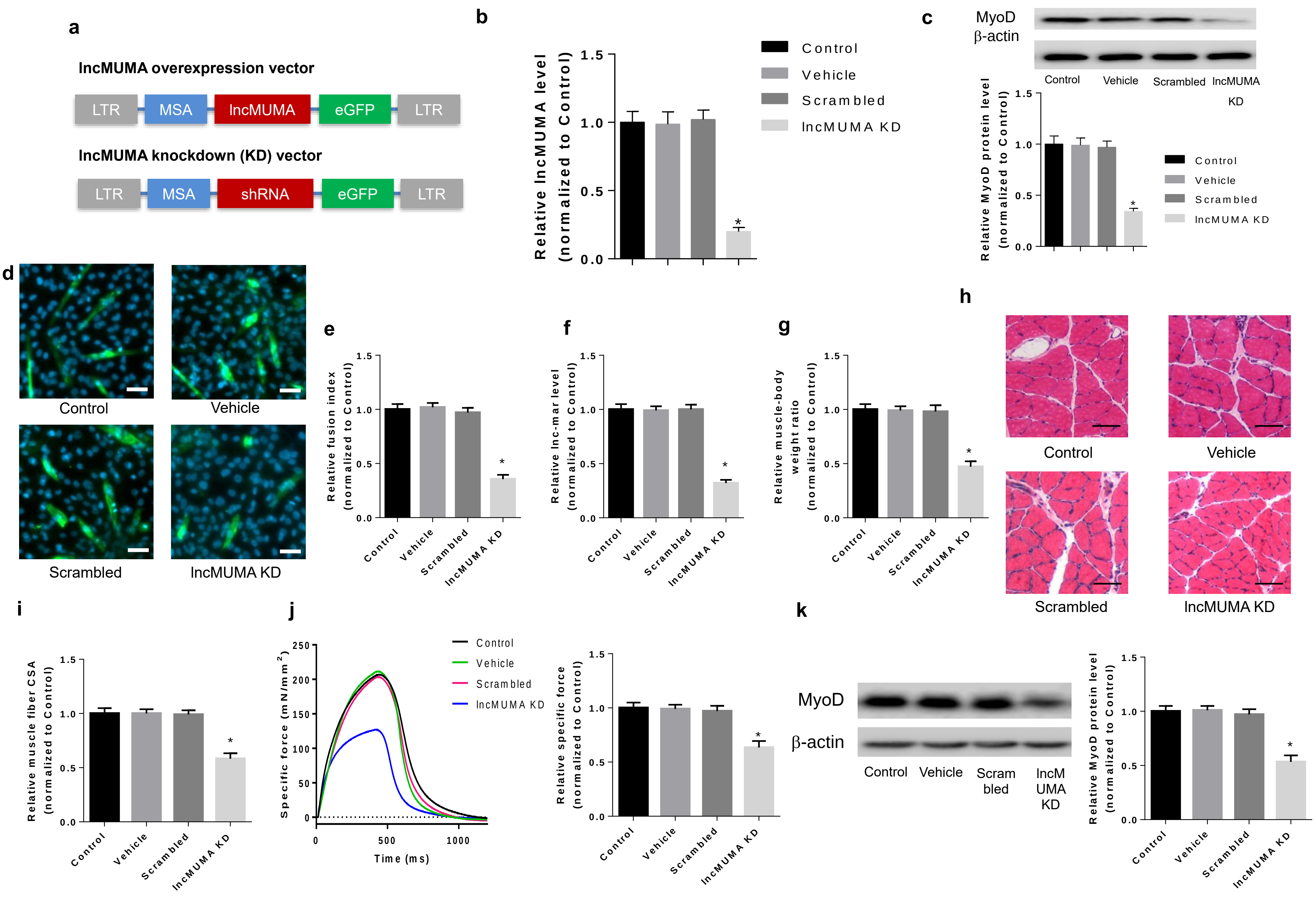
a
lncMUMA overexpression vector
LTR
MSA
lncMUMA
eGFP
LTR
lncMUMA knockdown (KD) vector
LTR
MSA
shRNA
eGFP
LTR
b
Relative lncMUMA level (normalized to Control)
Control
Vehicle
Scrambled
lncMUMA KD
c
MyoD
β-actin
Control
Vehicle
Scrambled
lncMUMA KD
Relative MyoD protein level (normalized to Control)
d
Control
Vehicle
Scrambled
lncMUMA KD
e
Relative fusion index (normalized to Control)
f
Relative lnc-mar level (normalized to Control)
g
Relative muscle-body weight ratio (normalized to Control)
h
Control
Vehicle
Scrambled
lncMUMA KD
i
Relative muscle fiber CSA (normalized to Control)
j
Specific force (mN/mm²)
Time (ms)
Relative specific force (normalized to Control)
k
MyoD
β-actin
Control
Vehicle
Scram bled
IncM UMA KD
Relative MyoD protein level (normalized to Control)

Figure S3 LncMUMA silencing decreased the myogenesis in C2C12 myoblasts *in vitro* and decreased muscle mass/structure/strength in adult mice. (a) Schematic of lncMUMA overexpression and knockdown lentiviral vector for viral preparation, respectively. (**b**) Real-time PCR analysis of lncMUMA levels in C2C12 cells transduced with lentivirus constructed with either scrambled shRNA or lncMUMA knockdown (KD) vector on Day 7 of differentiation under normal gravity culture environment. (**c**) Western blot analysis of MyoD protein level in lentivirus transduced C2C12 cells on Day 7 of differentiation under normal gravity culture environment. (**d**) Representative images of C2C12 cells transduced with lentivirus on Day 7 of differentiation under normal gravity culture environment. Myosin was labeled with green fluorescence, and the nuclei were labeled with DAPI. Scale bar=50 μm. (**e**) The fusion index in lentivirus transduced C2C12 cells on Day 7 of differentiation under normal gravity culture environment. (**f**) Real-time PCR analysis of lncMUMA level in soleus muscle of mice treated with lentivirus with EV and lncMUMA KD vector, respectively. (**g-i**) Gastrocnemius muscle-to-body weight ratio (**g**), cross-sections from mid-belly gastrocnemius muscle (**h**) and muscle fiber CSA (**i**) in mice treated with lentivirus with scrambled shRNA and lncMUMA KD vector, respectively. Scale bar= 50 μm. (**l**) *In vitro* muscle function testing of specific force in gastrocnemius muscle of mice treated with lentivirus with scrambled shRNA and lncMUMA KD vector, respectively. (**J**) Expression of MyoD protein in gastrocnemius muscle of mice treated with lentivirus with scrambled shRNA and lncMUMA KD vector, respectively. n=5 for *in vitro* and n=10 for *in vivo*. U6 small nuclear RNA is used as the endogenous control of lncRNA. β-actin is used as the endogenous control for protein. Data are presented as mean ± SEM. * $P<0.05$ vs. Control.

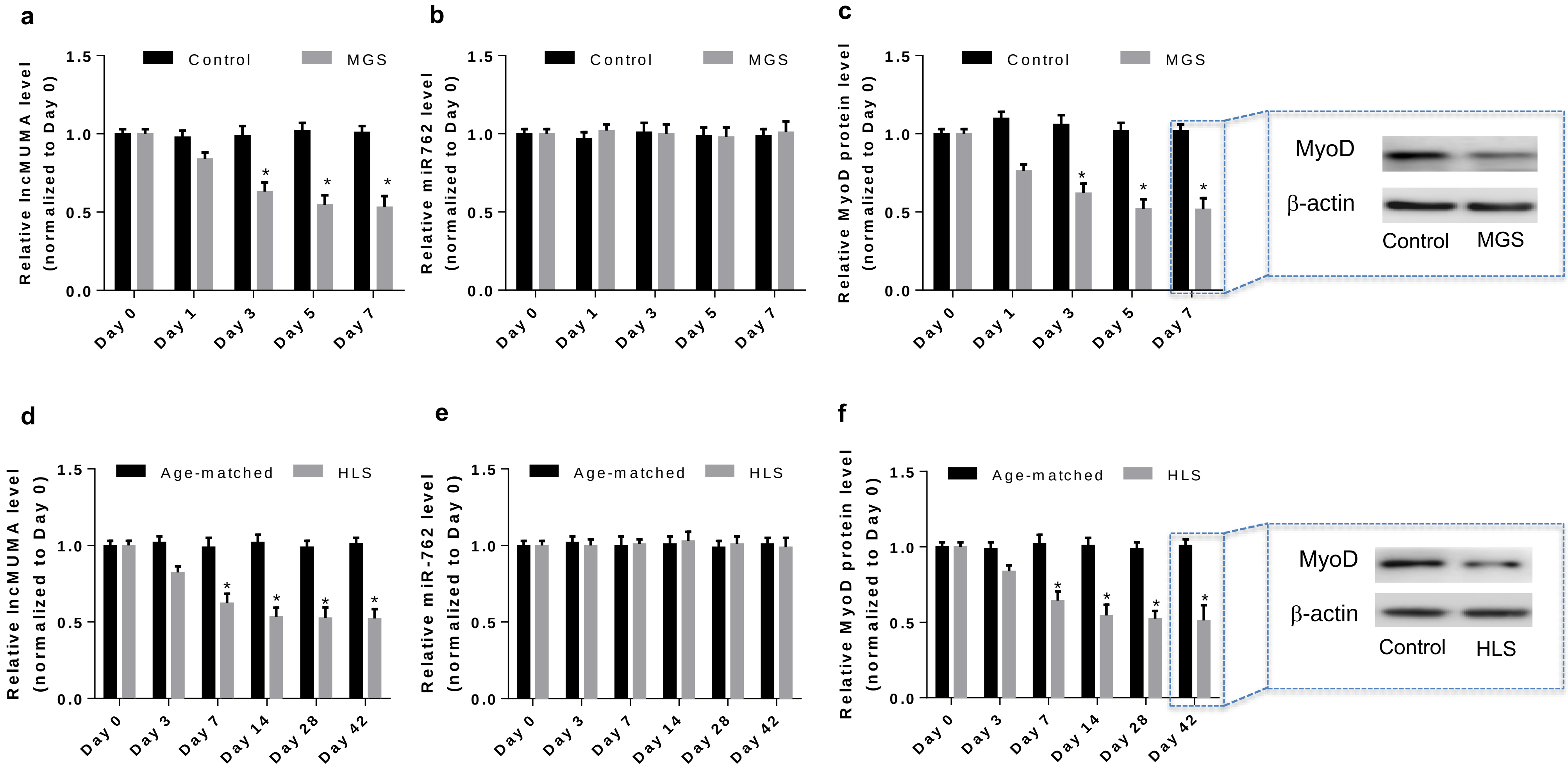

Figure S4 Reduced MyoD expression associated with decreased lncMUMA expression, without changes of miR-762, in microgravity-simulated (MGS) C2C12 myoblasts *in vitro* and in muscle tissues of hindlimb suspension (HLS) mice. (**a**) Real-time PCR analysis of lncMUMA levels in C2C12 cells with either normal gravity (Control) or MGS culture environment on Day 0, 1, 3, 5 and 7 of differentiation. (**b**) Expression level of miR-762 in C2C12 cells of each group at each time point. (**c**) (**left**) Expression level of MyoD protein in C2C12 cells of each group at each time point. (**right**) Representative western blot images of MyoD protein in C2C12 cells on Day 7 of differentiation. (**d**) Expression level of lncMUMA in gastrocnemius muscle of either healthy adult (Control) or HLS mice on Day 0, 3, 7, 14, 28 and 42 of HLS. (**e**) Expression level of miR-762 in gastrocnemius muscle of each group at each time point. (**f**)(**left**) Expression level of MyoD protein in gastrocnemius muscle of each group at each time point. (**right**) Representative western blot images of MyoD protein in gastrocnemius muscle of either Control or HLS mice on Day 42 of HLS. n=5 (*in vitro*) and 10 (*in vivo*) at each time point for each group. U6 small nuclear RNA is used as the internal control of lncRNA and miRNA. β-actin is used as the control for protein. Data are presented as mean ± SEM. *$P<0.05$ vs. Corresponding Day 0.

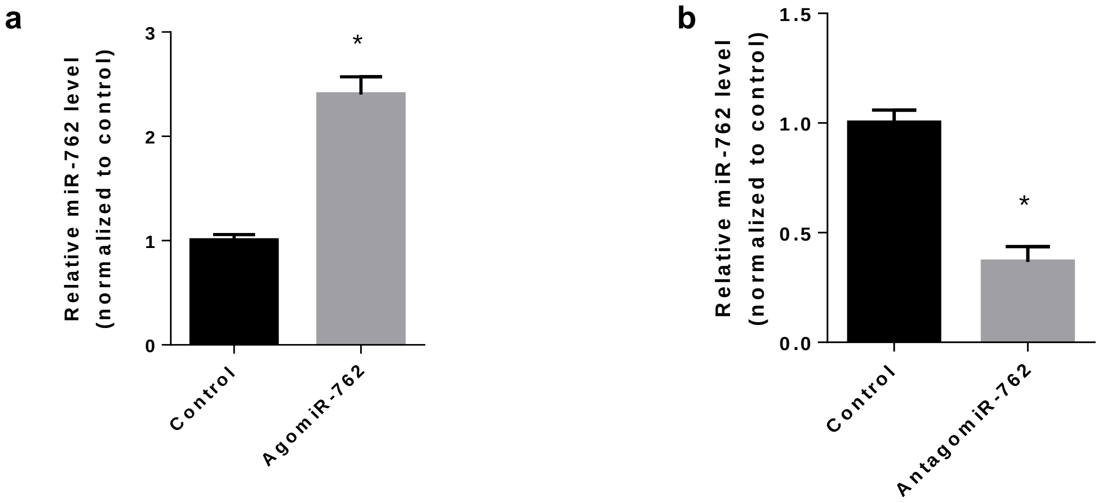

Figure S5 AgomiR-762 and AntagomiR-762 regulated the expression level of miR-762 in C2C12 myoblasts *in vitro*. Real-time PCR analysis of miR-762 level in C2C12 cells treated with AgomiR-762 (**a**) and AntagomiR-762 (**b**). U6 small nuclear RNA is used as the endogenous control. Data are presented as mean ± SEM. * $P<0.05$ vs. Control.

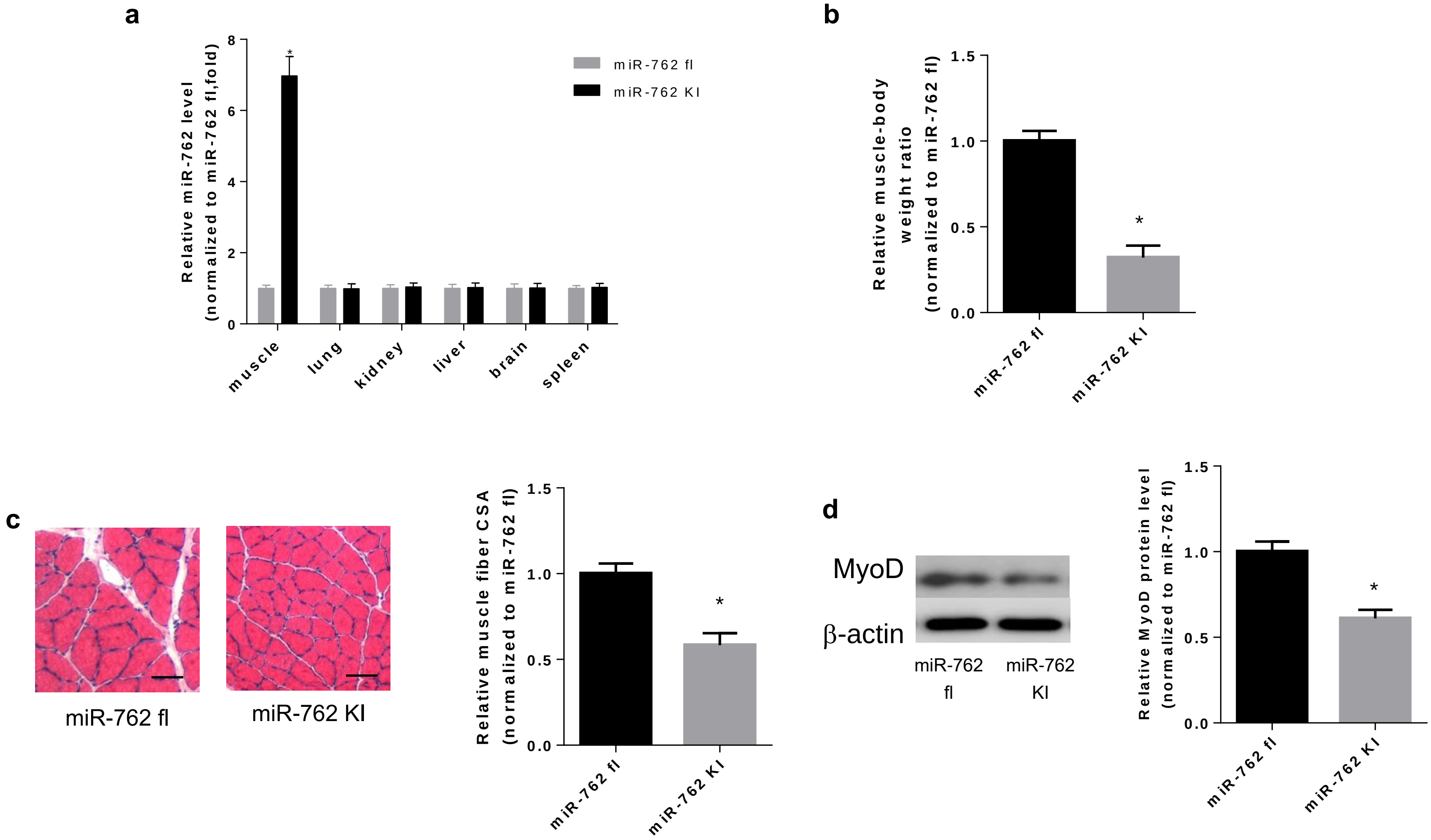

Figure S6 Phenotype analysis of MyoD expression, muscle mass/structure/function in muscle-specific miR-762 knockin mice. (a) Real-time PCR analysis showing the miR-762 expression in muscle, lung, kidney, liver, brain and spleen tissues isolated from ROSA26-PCAG-STOP^{flox}-*miR-762*-eGFP (miR-762 fl) and miR-762 knockin (miR-762 KI) mice, respectively. **(b)** Gastrocnemius muscle-to-body weight ratio in miR-762 fl and miR-762 KI mice, respectively. (**c**) Cross-sections from mid-belly gastrocnemius muscle and muscle fiber CSA in miR-762 fl and miR-762 KI mice, respectively. Scale bar= 50μm. (**d**) Expression level of MyoD protein in gastrocnemius muscle of miR-762 fl and miR-762 KI mice, respectively. n=10 for each group. U6 small nuclear RNA is used as the endogenous control of miRNA. β-actin is used as the endogenous control for protein. Data are presented as mean ± SEM. *P<0.05 vs. miR-762 fl group.

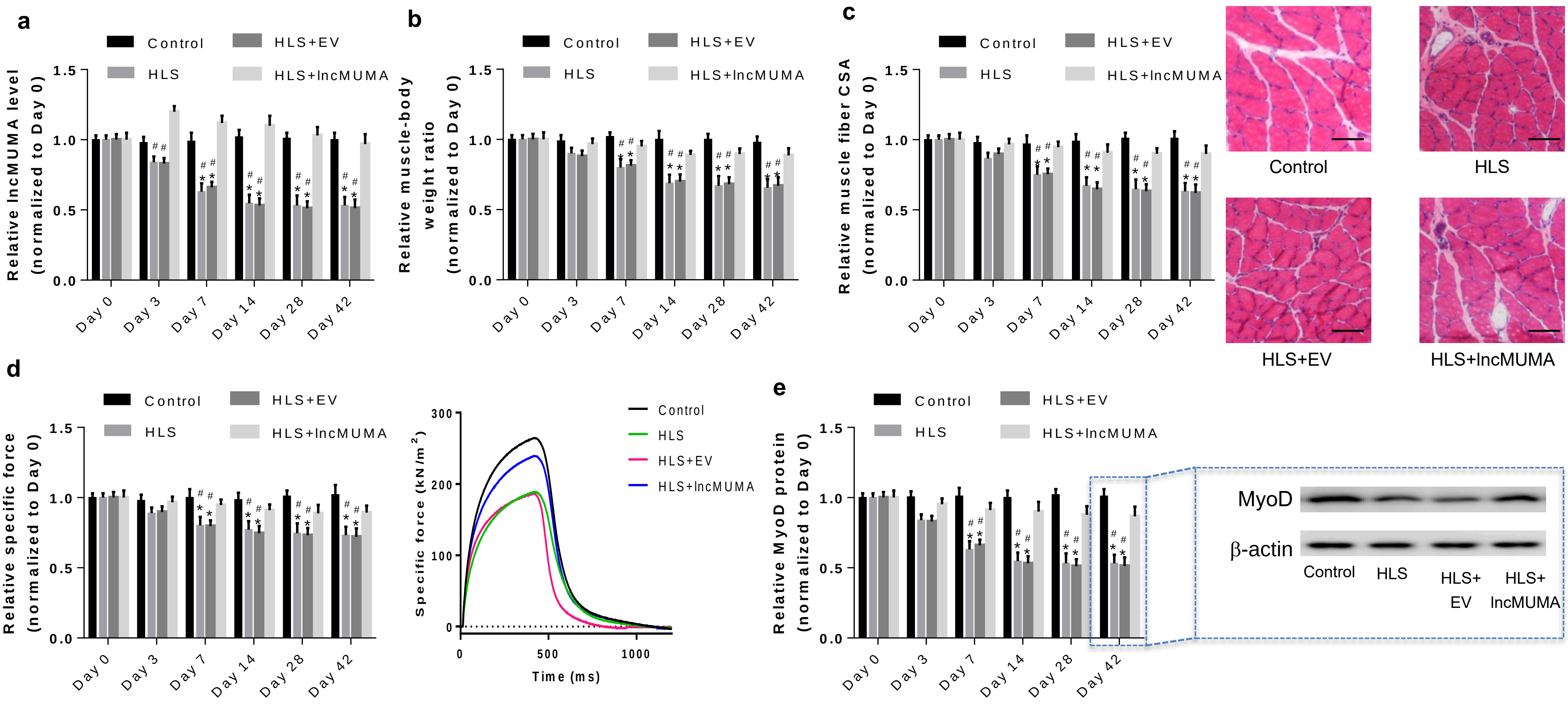

Figure S7 Skeletal muscle-specific overexpression of lncMUMA before unloading attenuated the decreases of MyoD, muscle mass/structure/function from baseline following mechanical unloading. (**a**) Real-time PCR analysis of lncMUMA levels in gastrocnemius muscle of normal control, hindlimb suspension (HLS), HLS+empty vector (EV) and HLS+lncMUMA vector (lncMUMA) mice, respectively, on Day 0, 3, 7, 14, 28 and 42 of HLS. (**b**) Gastrocnemius muscle-to-body weight ratio in each group at each time point. (**c**) (**left**) Gastrocnemius muscle fiber CSA in each group at each time point. (**right**) Cross-sections from mid-belly gastrocnemius muscle in each group at Day 42 of HLS. Scale bar= 50μm. (**d**) (**left**) *In situ* muscle function testing of specific force in gastrocnemius muscle of each group at each time point. (**right**) Specific force in gastrocnemius muscle of each group at Day 42 of HLS. (**e**) (**left**) Expression level of MyoD protein in each group at each time point. (**right**) Expression level of MyoD protein in gastrocnemius muscle of each group at Day 42 of HLS. n=10 at each time point for each group. U6 small nuclear RNA is used as the internal control of lncRNA. β-actin is used as the control for protein. Data are presented as mean ± SEM. * *P*<0.05 vs. Control, # *P*<0.05 vs. HLS+lncMUMA.

a

Sequence of lncMUMA-h in human

>NR_147193.1 Homo sapiens DM1 locus antisense RNA (DM1-AS), long non-coding RNA
GATTTGGGAAGGAGCTCGGAATGGAGCCGCTGGAAGAGGAGACGCGTGCGGGGAGAGGGCCC
GGGCGGGTGCCTGTCCCGTCCACACTTAGTCCCCGCGCCCCGCGGGCGCCTGAGATTGTGAG
CTGGTCCCGGGAGATGTCCGAGGACCTCGGCGCGCCGGCCCCAGCAGTGGGCACGGGGGAA
GAGGGCTGGTGGACGGGATGTCCCCGGGAGAGCTGGACTTGCGCCGCCCGAGGCCCCTCACC
TCTTGCAGGGCGCGCCGCCTCCGGCCCCGGTCCGGTCGCGCTGTCGCCGGTTCTTGAACCAG
TTGCTGACCTGCGTGAGCGACAGGCCGGTGTCTATACACGCCCCGCGGAGCAGACGGCCCACC
TCCTCCCGGTCCTCCGGGGAAGGGGACACATGAGGGACTCACCTGTGGCTCCCTCTGCCTGCA
GCAACTCCATCCGCTCCTGCAACTGCCGGACGTGTGCCTCTAGGTCCCGGTTCCGAGCCTCTG
CCTCGCGTAGTTGACTGTGGGGAGGTAAGGACGGTGAGTCCGTCCGGGCCGGACGAGAGGGG
ATGCCAAGGGTTGCCACCGGCCCGCATCCCGGCCCCGGCCCCGGCCCCGATCCCGACCTGGC
GAAGTTCTGGTTGTCCGTGCGGATGGCCTCCATCTCCCGGCTCAGGCTCTGCCGGGTGAGCAC
CTCCTCCTCCAGGGCTTCCTGGAGCTCCCGCAGCGTCACCTCGGC

b

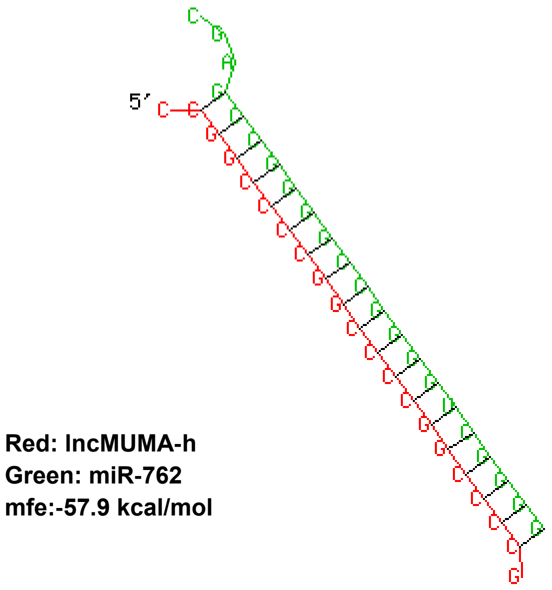

c

lncMUMA-h-WT 5' cCGGCCCCGGCCCCGGCCCCg 3'
| | | | | | | | | | | | | | | | | | | |
miR-762-WT 3' cgaGCCGGGGCCGGGGUCGGGG 5'
lncMUMA-h-Mut 5' cCGUAUAAAUGAGUUAGGCCg 3'
miR-762-Mut 3' cgaGCGAUAUAAACACAAUUGG 5'

d

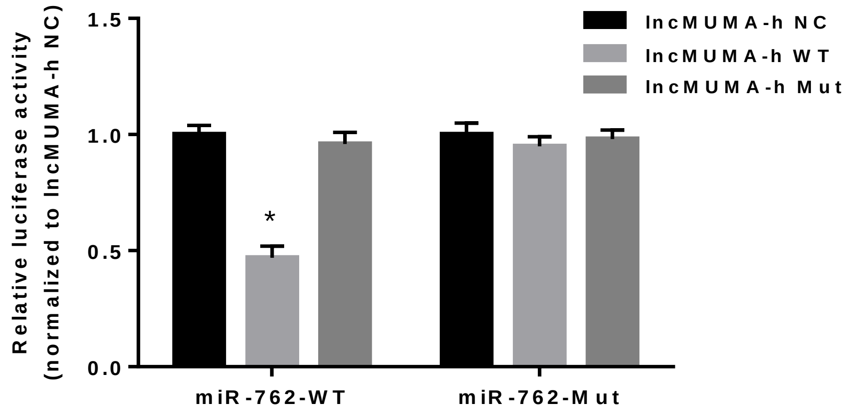

Figure S8 lncMUMA was functionally conserved in primary human skeletal muscle cells (pHSkMC). (**a**) Sequence of lncMUMA in human (lncMUMA-h). (**b**) Bioinformatic prediction of miR-762 as a target miRNA of lncMUMA-h by RNAhybrid 2.12. mfe: minimum free energy. (**c**) Sequence of wild type and mutated binding site between miR-762 and lncMUMA-h. (**d**) Luciferase reporter assay of either wild type miR-762-transfected (miR-762-WT) or mutated miR-762-transfected (miR-762-Mut) pHSkMCs from healthy adults treated with negative control (lncMUMA-h NC), wild type binding site of lncMUMA (lncMUMA-h WT) and mutated binding site of lncMUMA (lncMUMA-h Mut), respectively. n=3. * *P*<0.05 vs. lncMUMA-h NC. Data are presented as mean ± SEM.

Table S2 Primer sequences of lncRNA, miRNA and mRNA

Gene	Primer sequences
lncMUMA	forward: TTGTACTTCAGCTCCCAGGG<br>reverse: CCAGCGACTCACTAGATGGT
miR-762	forward: ACACGGGGCTGGGGCCGGGGCCGAGCGCCTC<br>reverse: CTCAGGGGCTGGGGCCGGGGCCGAGCCAGA
Myod1	forward: AGTGAATGAGGCCTTCGAGA<br>reverse: GCATCTGAGTCGCCACTGTA
